# Effectiveness of supervised vs brief counselling physical activity promotion interventions in breast cancer survivors on aromatase inhibitors: the PAC-WOMAN randomized controlled trial

**DOI:** 10.1186/s12966-026-01873-5

**Published:** 2026-01-22

**Authors:** Eliana V. Carraça, Bruno Rodrigues, Sofia Franco, Inês Nobre, Flávio Jerónimo, Vítor Ilharco, Pedro F. Silva, António L. Palmeira, Marlene N. Silva

**Affiliations:** 1 CIDEFES, FEFD, Universidade Lusófona, Lisbon, Portugal; 2 CIFID2D, Universidade Do Porto, Porto, Portugal; 3 Faculdade de Motricidade Humana, Universidade de Lisboa, Cruz Quebrada, Portugal; 4 IADE, Universidade Europeia, Porto, Portugal

**Keywords:** Survivorship, Aromatase Inhibitors, Quality of Life, Physical Activity, Active Lifestyle, Brief counseling, Self-Determination Theory

## Abstract

**Background:**

Aromatase inhibitors (AI) are used to treat hormone-receptor-positive breast cancer, but have several side effects that often lead to premature discontinuation/switch. Physical activity (PA) can attenuate side effects and improve quality of life. However, most cancer survivors fail to meet PA guidelines. Theory-based PA interventions are effective in breast cancer survivors, but scarce. PAC-WOMAN tested the effects of two PA promotion interventions (supervised exercise vs brief counseling) on primary outcomes – PA, sedentary behavior, quality of life –, and secondary outcomes – body composition, fitness, physical function, sleep, body image, depression, psychological well-being, pain, and motivational variables – in women on AI.

**Methods:**

This pragmatic randomized controlled trial included 110 women with stage I-III hormone-receptor-positive breast cancer on AI (56.1 ± 7.6 yr; 28.1 ± 5.9 kg/m^2^; 23.4 ± 20.1 months on AI), randomized to: 1) brief PA counseling (PAC); 2) structured exercise (ExG); 3) waiting-list control. Primary and secondary outcomes were evaluated with validated instruments at the end of the intervention period. Repeated measures’ Anovas, adjusted for age, AI duration, body mass index and chronic diseases, were conducted. Bonferroni corrections were applied (significance level at *p* < 0.0083).

**Results:**

Significant group-by-time interactions were observed for moderate-vigorous PA (MVPA), exercise motivations and affect, muscle strength and leg endurance (*p* ≤ 0.008). Objectively measured PA showed no significant effects in either intervention group, except for a near-significant MVPA increase of 107 min/week [95% CI: 4; 209] over time in ExG. Larger improvements were observed in all self-reported PA indicators in ExG, while PAC revealed gains only in light PA and active choices. ExG improved global health status, physical functioning and physiological indicators (e.g., fitness, body composition) over time; PAC enhanced future perspectives, body image functioning, breast symptoms, and other psychological outcomes, with changes in depressive symptoms (-1.4 [-2.4; -0.3]) and life satisfaction (0.8 [0.2; 1.5]) approaching significance.

**Conclusions:**

PAC-WOMAN showed that ExG and PAC yielded distinct benefits for breast cancer survivors on AI. ExG improved total PA, fitness, physical function, physical quality of life, and alleviated pain symptoms. PAC primarily enhanced light PA and psychological outcomes such as body image and life satisfaction. Together, these findings highlight the potential scalability of PAC alongside the robust physical benefits of ExG.

**Trial registration:**

April 2023 – NCT05860621; https://doi.org/10.17605/OSF.IO/ZAQ9N; UMIN000050945.

**Supplementary Information:**

The online version contains supplementary material available at 10.1186/s12966-026-01873-5.

## Background

Breast cancer is one of the three most common cancers worldwide [1]. Hormone-receptor-positive breast cancer is diagnosed in 75% of the cases and is often treated with aromatase inhibitor hormonal therapy (AI), after primary treatment completion, in post-menopausal women [2]. AI improve disease-free survival by 10–40% [3], but have multiple detrimental side effects (e.g., arthralgia, osteoporosis) that affect breast cancer survivors’ quality of life [4] and result in premature AI discontinuation/switch, lower treatment efficacy and increased mortality [5]. There are currently no behavioral interventions effective in the promotion of hormonal therapy adherence [6], although physical activity can be a promising way to increase adherence to AI [7–9].

There is compelling evidence supporting the beneficial role of physical activity (PA) in cancer survivors (i.e., from diagnosis to the end of life). PA effectively improves physical fitness, physical functioning, sleep, body image, and quality of life, and decreases treatment-related adverse effects (e.g., fatigue, pain), cancer recurrence and mortality [10, 11]. PA also reduces AI associated joint symptomatology and risk of osteoporosis, fracture, cancer recurrence or death [12, 13]. However, most cancer survivors fail to meet established PA guidelines [14].

Supervised exercise programs offer significant benefits for patients living with or beyond cancer [10, 15], especially in the short-term [16], however they are expensive and labor intensive, preventing their full integration in the health care system [17]. Furthermore, these programs do not generally stimulate patients’ responsibility for their own change, endangering the sustainability of PA behaviors and associated health outcomes. Thus, effective and scalable interventions to enhance the adoption/maintenance of regular PA are required.

Theory-based interventions, delivered in a need-supportive way and using behavior change techniques (e.g., self-monitoring, adding objects to the environment, goal setting or action planning) known to be linked to long-term PA outcomes [18, 19], to foster self-determined choices and the development of self-regulatory skills, seem to increase PA and well-being in general populations [20, 21] and breast cancer survivors [19, 22]. Among these, brief counseling interventions, involving an approach to patient's motivations, barriers, preferences, and opportunities to perform PA [23], have led to clinically relevant increases in PA in the general population [24], and suggested to be as effective as more intensive interventions [25]. Brief counseling interventions might thus be a practical alternative to enhance PA sustained adoption in real-world health care or community settings. Still, brief counseling interventions targeting cancer survivors remain limited and their effectiveness unknown.

The PAC-WOMAN trial was designed to test the effectiveness of two types of interventions (structured exercise program vs. brief theory-based PA counseling) against a waiting-list control group, immediately post-intervention and in the long-term, on the promotion of sustained changes in PA, sedentary behavior, and quality of life, in breast cancer survivors on AI. This paper describes post-intervention (4-month) effects of the PAC-WOMAN trial on its primary (PA, sedentary behavior, and quality of life) and secondary outcomes at the physical (e.g., strength, cardiorespiratory fitness) and psychosocial level (e.g., body image, motivations).

## Methods

A comprehensive description of the study protocol, including recruitment procedures, eligibility criteria, randomisation process, interventions, and full assessments, is described in detail elsewhere [26]. The trial was registered at www.clinicaltrials.gov (NCT05860621), received ethical approval, and was conducted according to the Helsinki declaration [27].

### Study design and setting

PAC-WOMAN is a three-arm pragmatic randomized controlled trial (RCT), comprising a 4-month intervention and a 12-month follow-up, aimed at promoting long-term active lifestyles and quality of life, albeit with different approaches (brief PA counseling vs. supervised structured exercise), compared to a waitlist control. This trial was conducted under real world circumstances at local gyms from a main urban center, served by several hospitals. Participants entered the study in three annual cohorts, beginning around January/February. Due to recruitment and timeline constraints, some cohorts started with fewer participants than others (cohort 1: 31 participants; cohort 2: 56; cohort 3: 25). After eligibility screening, informed consent and baseline assessments, participants were randomized, using an automated computer-generated randomization scheme performed by a researcher not in direct contact with them. Both participants and staff were blinded to group allocation at the time of recruitment and baseline assessments, but behavior change interventions prevented any concealment after randomization. The CONSORT checklist can be found in Additional File 1.

### Participants

Participants were recruited by medical referral from main hospitals with oncology centers from December 2021 to November 2023. Additional, more direct, strategies (e.g., contacting patient associations, posting Facebook adds; requesting public figures to disseminate the study, distributing flyers in cancer-related sport events), were also employed.

To be included, participants had to be post-menopausal women (< 70 years), with stage I-II hormone-receptor-positive breast cancer, on AI after primary treatment (≥ 1 month), an ECOG 0–1. Participants were ineligible if they had stage IV cancer or synchronous tumors, uncontrolled hypertension or cardiac/pulmonary disease, medical contraindications to exercise, and were unable to provide informed consent or follow the program schedule.

Sample size calculations for the primary outcomes, considering a 25% dropout, indicated that 98 participants were required to detect a small to moderate effect size (α = 0.05; statistical power = 0.80) using G*Power 3.1.

**Fig. 1:**
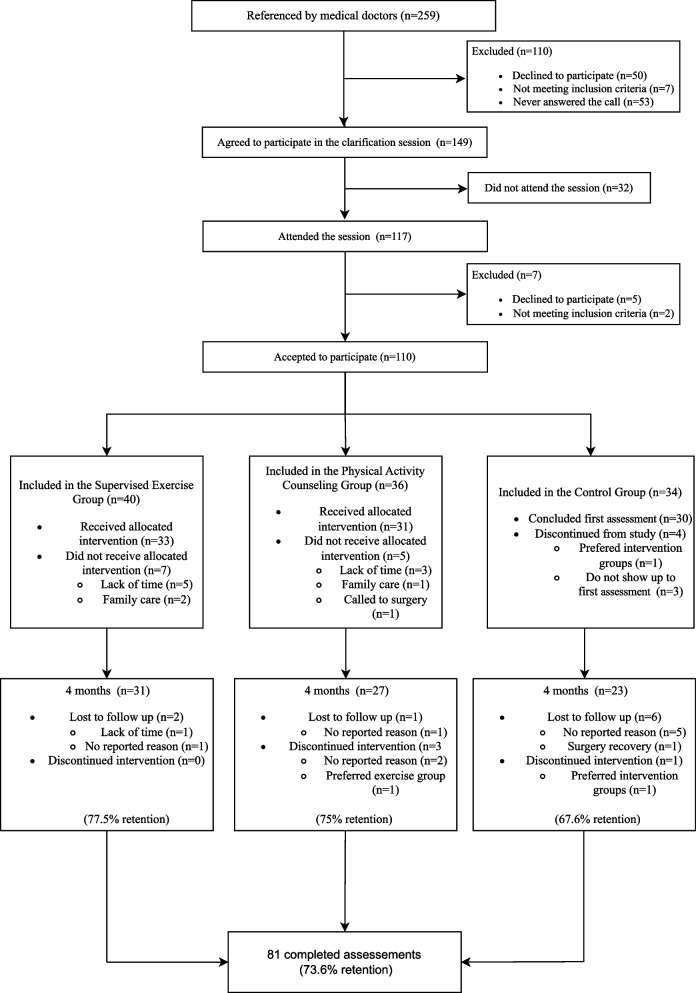
PAC-WOMAN trial flowchart

All participants provided written informed consent prior to participation in the trial and were informed that they could withdraw at any time. After baseline assessments, they were randomly allocated to brief PA counseling (PAC), supervised structured exercise (ExG), or waitlist control (CG). Participants’ flow in the study is depicted in Fig. 1.

### Interventions

The PAC program comprised 8 group-based sessions throughout the 4-month period. It was designed according to Self-Determination Theory (SDT) basic tenets [28], employing evidence-based, theoretically driven behavior change techniques, to empower participants to develop self-regulation resources and autonomous motivations to integrate PA into their daily lives. Sessions and intervention materials supported autonomy, competence, and relatedness basic psychological needs, using strategies such as providing choice, meaningful rationales, linking participants’ health behavior and core values, goal setting, action planning, self-monitoring, coping planning, and weighing pros/cons of change in coherence with life values/priorities. A Xiaomi Mi Band 5 activity tracker was provided to participants to encourage self-monitoring. Exercise professionals, trained in brief counseling skills for health behavior change, delivered the sessions in an exercise setting. A participants’ manual and a home-based exercise booklet were offered to participants [29]. See Additional File 2 for more detailed information.

The ExG program was designed according to the current guidelines for exercise prescription and safe practice in cancer populations [10, 30, 31], and consisted of two supervised, tailored group sessions/week over 4 months, with progressive intensity and complexity, plus a thematic group class every 15 days, allowing them to experience different physical activities. Each sessions, led by qualified exercise professionals, included: (1) a 10–30’ warm-up (mobility exercises and body weight activities); (2) a 30–45’ strength component (6–7 large muscle group exercises at 60–80% of one maximal repetition, 10–15 repetitions, 2–3 sets); (3) a 20–30’ aerobic training component (at 40–60% of reserve heart rate), performed in a self-selected apparatus (e.g., treadmill, bike); and (4) a 5’ cool-down. (check Additional File 2 for more detailed information). Individual target zone intensities were set, based on baseline assessments and existing guidelines [10, 32], using the Heart Rate Reserve (HRR) method and the age-predicted Tanaka formula for Maximal Heart Rate. Strength training zones were based on the One-Repetition Maximum (1-RM), estimated with the Baechle and Groves’ coefficient [33], following the 10-Repetition Maximum test for chest press, leg press, and horizontal row machines. Target zones were monitored throughout the sessions, and exercise prescriptions adapted to participants’ needs.

Participants allocated to the waitlist control group were asked to continue their daily routines, receiving their standard medical care. After the final follow-up assessment, they received a combined program, including supervised exercise sessions and PAC contents.

### Fidelity and training of intervention facilitators

Both interventions across cohorts were delivered in the same exercise setting (health club) by the same implementers to ensure fidelity. Only certified exercise physiologists (with master's degrees in exercise and health), trained in brief counselling and self-regulatory skills’ facilitation, were selected to implement the programs. In addition, an implementers’ manual, mock delivery training sessions, and a session logbook were used to standardize delivery and document whether all components were delivered according to the plan (and if not, why, what adaptations were made). Minimal deviations to the protocol were registered.

### Assessments

Participants were assessed at baseline and 4 months (intervention’s end), in standardized conditions, in a calm comfortable environment.

### Primary outcomes

Physical activity and sedentary behavior. Objective measures of PA were obtained through Actigraph GT9X wrist accelerometers. Participants were asked to wear the devices continuously for 8 days during their daily activities and sleep. Data were included only if participants had at least four valid days (≥ 2 two weekdays and ≥ 1 weekend day), with ≥ 960 min/day of wear time [34]. Counts were sampled in 1 s epochs to ensure sufficient sensitivity for low intensity activities, and categorized as sedentary, light, moderate and vigorous, using Montoye’s cut-points [35]. These are validated for wrist placement and this device [35] and are preferred over Freedson’s cut-points developed for waist-worn monitors. Outcomes were expressed as weekly minutes of light, moderate, and vigorous PA, and daily hours of sedentary behavior.

The Short-Form (9-item) International Physical Activity Questionnaire (IPAQ) assessed self-reported PA and sedentary behavior, capturing weekly frequency and duration of light, moderate, and vigorous PA and sitting time on week and weekend days. Weekly minutes of total PA, light PA (LPA), moderate-vigorous PA (MVPA), and total sitting hours/week were derived from the collected data [36]. The 6-item Activity Choice Index (ACI) [37] measured everyday physically active choices made over the past month (e.g., “using stairs instead of escalators or lifts”). Cronbach’s alpha was 0.79.

Quality of life. The European Organization for Research and Treatment of Cancer Quality of Life Questionnaire Core 30 (EORTC QLQ-C30) and its breast cancer module (EORTC QLQ-BR23) measured quality of life [38]. EORTC QLQ-C30 comprises 30 items, organized in 8 multi-item functional (physical, role, emotional, cognitive, and social) and symptom (fatigue, pain, and nausea) subscales, one global health status subscale, and 6 single items (dyspnea, insomnia, appetite loss, constipation, diarrhea, and financial difficulties). Of these, subscales assumed to be less affected by AI were not included (nausea, dyspnea, appetite loss, constipation, and diarrhea). EORTC QLQ-BR23 (23 items), organized into 4 functional and 4 symptom breast cancer-related subscales, measured body image, sexual functioning, future perspectives, systemic therapy side effects, breast symptoms, and arm symptoms. Five additional items from EORTC QLQ-BR45 [39] assessed musculoskeletal symptoms (joint, bone, and muscle pain/discomfort).. Cronbach’s alpha ranged between 0.65 and 0.92.

### Secondary outcomes (physical measures)

Anthropometric and body composition measures. Body weight was measured with a digital scale (SECA, Germany) and height with a balance-mounted stadiometer. Body mass index in kilograms per square meter was calculated from weight (kg) and height (m). Waist circumference was measured according to the NIH protocol [40]. Bioelectrical Impedance (Impedimed, Australia) was used under standardized conditions, by experienced technicians to assess percent body fat.

Cardiorespiratory fitness. A submaximal, 8-min, single-stage treadmill walking test, comprising a 4-min warming up at a self-selected speed (at 50–70% age-predicted maximal heart rate), and 4 additional minutes at a 5%-increased workload, was performed to measure cardiorespiratory fitness [41]. VO_2_​max was estimated using Ebbeling’s regression Eq. [41]: $$\lbrack\mathrm{VO}2\max(\mathrm{mL}\cdot\mathrm{kg}-1\cdot\min-1)=15.1+21.8\times\mathrm{Speed}$$$$-0.327\times\mathrm{HRss}-0.263\times\mathrm{Speed}\times\mathrm{Age}+0.00504$$$$\times\mathrm{HRss}\times\mathrm{Age}+5.98\times\mathrm{Gender}\rbrack$$.

Muscle strength. Handgrip and maximal strength tests assessed muscular strength. Participants were instructed to hold the JAMAR handgrip with their maximal strength. Maximal muscular strength was determined for chest press, horizontal seated row and leg press, using a 10-repetition maximum test [42].

Physical function. The Timed Up and Go test measured functional mobility, by assessing the time a person took to rise from a chair, walk 2.44 m, turn around, return, and sit down [43]. The Sit to Stand test assesses leg strength and endurance by counting chair stands with arms crossed over the chest completed in 30 s [44]. The Stand on one foot test measured balance by instructing participants to stand on one foot with their eyes open (both sides were tested) for a maximum of 20 s and having their time recorded [45].

Upper limb flexibility. Linear measures were taken using the Back Scratch protocol [46] to measure flexibility on both shoulders with a SECA measuring tape.

Sleep duration. Actigraph GT9X wrist accelerometers assessed sleep duration (hours/day), applying the criteria described above for PA. Raw data were reintegrated into 60-s epochs, and sleep periods were identified using the Cole- Kripke algorithm [47].

### Secondary outcomes (psychosocial measures)

Psychological well-being indicators. Four items Four items were used to measure satisfaction with life, optimism, and purpose of life and daily activities [48].

Depression. The 7-item depression subscale from the Hospital Anxiety and Depression Scale [49] measured depression (Cronbach’s alpha = 0.86).

Body image. The 10-item Body Image Scale [50] assessed participants’ affective (e.g., feeling self-conscious), behavioral (e.g., difficulty at looking at the naked body), and cognitive (e.g., satisfaction with appearance) dimensions of body image (Cronbach’s alpha = 0.92).

Pain severity and interference. Two items from the Brief Pain Inventory were used to assess pain severity “on average” and “right now” [51]. The 7-item Pain Disability Index [52] measured the interference of pain on routine activities and functioning like family and home responsibilities, recreation, social activity, occupation, self-care activities (Cronbach’s alpha = 0.82).

Sleep quality. Six items from the Pittsburgh Sleep Quality Index [53] were used to measure sleep disturbance components of sleep quality (Cronbach’s alpha = 0.70).

Basic psychological needs satisfaction/frustration. The 24-item Basic Psychological Need Satisfaction and Frustration Scale [54] assessed satisfaction/frustration of autonomy, competence, and relatedness for exercise. Global need satisfaction and frustration scores were calculated by summing the autonomy, competence and relatedness satisfaction or frustration subscales, respectively (Cronbach’s alpha above 0.82).

Exercise motivations. The 18-item Portuguese version of the Behavioral Regulation in Exercise Questionnaire-3 [55] measured amotivation, external regulation, introjected regulation, identified regulation, integrated regulation, and intrinsic motivation (Cronbach’s alpha between 0.64 and 0.82). A global score for autonomous motivations was calculated by adding up the intrinsic, integrated and identified regulations’ subscales.

Exercise self-efficacy. The 9-item Modified Bandura’s Exercise Self-Efficacy Scale [56] measured perceived capacity to perform exercise under different conditions (Cronbach’s alpha = 0.83).

Affective response to exercise. The affective valence of exercise was measured with the 11-point Feeling Scale [57], ranging from −5 (“Very bad”) to + 5 (“Very good”).

### Other relevant information

Demographic information including age, time since diagnosis, AI therapy duration, cancer stage and treatment, marital status, education, profession, financial situation, and presence of chronic diseases, were collected to characterize participants. Attendance to exercise and PAC sessions and compliance with prescribed exercise doses (achieved, exceeded or remained below) were also registered.

## Adverse events

A simplified version of the Common Terminology Criteria for Adverse Events (CTCAE v.5), using its broader categories (i.e., cardiac, respiratory, nervous system, infectious, skin, musculoskeletal/connective tissue, and immune system disorders) was used at each timepoint to assess the occurrence, frequency, and severity of any adverse events in the previous 3 months.

### Statistical procedures

Split-plot repeated-measures Anovas, adjusted for age, AI therapy duration, BMI and presence of chronic diseases (operationalized as a dummy variable with a yes/no answer format), were conducted for primary and secondary outcomes, using an intent-to-treat approach. Missing data were handled with multiple imputation using prediction-matrix techniques and 20 imputed datasets [58]. Bonferroni-corrected pairwise comparisons [k = (a)*(a-1)/2 possible pairs, where a = number of treatments] were performed to further explore group and time differences. Considering that there were three intervention groups (k = 3) and two time points (k = 3*2 = 6), a p-value < 0.0083 (0.05/6) was considered significant. Effects/differences with p-values between 0.05 and 0.0083 were interpreted as near/approaching significance to highlight important trends and facilitate interpretation of group by time interactions. Eta-squared (Eta^2^) was calculated to quantify effect sizes (0.01 = small, 0.06 = moderate, 0.14 = large) [59].

## Results

A total of 259 breast cancer survivors were referred by medical doctors. Of these, 50 women (19%) declined to participate for various reasons (i.e., lack of time, incompatible schedules, high family demands, residing far from the program location, commuting difficulties, health issues), 53 (20%) did not respond to our contacts and 7 (3%) did not meet eligibility criteria. Of 149 women (58%) who agreed to participate in clarification sessions, 117 (79%) attended. Of these, five declined to participate (due to incompatible schedules and living far from the implementation site) and two did not meet the inclusion criteria. Therefore, 110 participants were enrolled in the study and subsequently randomized, as depicted in Fig. 1.

Of the 40 participants initially allocated to the ExG, 33 received the intervention (see reasons for early dropout in Fig. 1), and 31 completed the 4-month assessment (77.5% retention). Attendance to the program varied (> 75%: *n* = 14, > 45%: *n* = 23, < 25%: *n* = 1), though compliance with prescribed aerobic and strength doses was high for all but three participants. Intervention-related adverse events were few and included mild chest muscle soreness (n = 5) and edema (n = 2). Participants with prior lymphedema, chronic knee pain or back injuries (*n* = 5) had acute swelling and pain episodes, prompting exercise prescription adjustments. Adverse events unrelated to the intervention included a shoulder injury (*n* = 1), sprained ankles (*n* = 2), peripheral neuropathy (*n* = 1), vomiting/nausea (*n* = 2), food poisoning (*n* = 1), COVID-19 infection (*n* = 2), and flu episodes (*n* = 2).

Of the 36 participants initially allocated to PAC, 31 received the intervention and 27 completed the 4-month assessment (75% retention). All 27 participants attended at least 5 of 8 sessions. Program-related adverse events were rare and mild – including cervical pain while executing some of the booklet exercises (*n* = 1), chest/breast burning sensation (*n* = 1) and increased low-back pain (*n* = 1) – and did not limit participation. Most adverse events were unrelated to the program: covid/flu (*n* = 3), pain in different body regions (*n* = 4), hypertension spikes (*n* = 1), hospitalization due to pre-existing inflammatory disease (*n* = 1).

Thirty-four participants were initially allocated to CG, but only 23 completed 4-month assessments (67.6% retention).

Overall, 81 participants completed assessments at interventions’ end (73.6% global retention).

Baseline characteristics are depicted in Table 1. No differences were found between groups for demographic, PA and quality of life measures.

**Table 1:** Baseline sample characteristics

	All (*n* = 110)	ExG (*n* = 40)	PAC (*n* = 36)	CG (*n* = 34)	Group differences (*p*-value)
Age (years)	56.22 ± 7.74	55.90 ± 6.91	56.44 ± 7.90	56.35 ± 8.65	0.948
Body mass index (kg/m^2^)	27.82 ± 4.79	28.18 ± 4.86	28.17 ± 5.12	26.79 ± 4.17	0.422
Time since diagnosis (months)	34.56 ± 21.44	36.75 ± 23.57	35.69 ± 23.81	30.79 ± 15.42	0.473
AI Therapy duration (months)	21.83 ± 17.14	22.30 ± 16.63	22.58 ± 19.88	20.47 ± 14.89	0.842
Marital status					0.174
Married	69 (62.7%)	22 (55.0%)	24 (66.7%)	23 (67.6%)	
Divorced	16 (14.5%)	6 (15.0%)	6 (16.7%)	4 (11.8%)	
Widow	5 (4.5%)	0 (0.0%)	2 (5.6%)	3 (8.8%)	
Single	20 (18.2%)	12 (30.0%)	4 (11.1%)	4 (11.8%)	
Educational level					0.244
Incomplete primary school	4 (3.6%)	1 (2.5%)	2 (5.6%)	1 (2.9%)	
Primary school	16 (14.5%)	4 (10.0%)	9 (25.0%)	3 (8.8%)	
9º grade	15 (13.6%)	5 (12.5%)	6 (16.7%)	4 (11.8%)	
High school	19 (17.3%)	11 (27.5%)	2 (5.6%)	6 (17.6%)	
Bachelor	56 (50.9%)	19 (47.5%)	17 (47.3%)	21 (58.8%)	
Profession					0.881
Full-time	57 (51.8%)	23 (57.5%)	18 (50%)	16 (47.1%)	
Part-time	10 (9.1%)	4 (10.0%)	3 (8.3%)	3 (8.8%)	
Unemployed	9 (8.2%)	4 (10.0%)	3 (8.3%)	2 (5.9%)	
Housewife	6 (5.5%)	2 (5.0%)	3 (8.3%)	1 (2.9%)	
Retired	19 (17.3%)	5 (12.5%)	5 (13.9%)	9 (26.5%)	
Medical leave	7 (6.4%)	1 (2.5%)	3 (8.3%)	3 (8.8%)	
Other	2 (1.8%)	1 (2.5%)	1 (2.8%)	0 (0.0%)	
Financial situation					
Hard	15 (13.6%)	6 (15.0%)	3 (8.4%)	6 (17.6%)	0.054
Sufficient for needs	54 (49.1%)	22 (55.0%)	23 (63.9%)	9 (26.5%)	
Comfortable	40 (37.3%)	12 (30.0%)	10 (27.8%)	19 (55.8%)	
Presence of chronic diseases					0.020
Yes	66 (60%)	29 (72.5%)	23 (63.9%)	14 (41.2%)	
No	44 (40%)	11 (27.5%)	13 (36.1%)	20 (28.8%)	
Cancer stage					0.993
Stage 1	16 (14.5%)	5 (12.5%)	6 (16.7%)	5 (14.7%)	
Stage 2	73 (66.3%)	28 (70.0%)	22 (61.1%)	23 (67.6%)	
Stage 3	21 (19.1%)	7 (17.5%)	8 (22.2%)	6 (17.6%)	
Cancer treatment					
Chemotherapy	77 (70.0%)	26 (23.6%)	28 (25.5%)	23 (20.9%)	0.449
Radiotherapy	91 (82.7%)	32 (29.1%)	32 (29.1%)	27 (24.5%)	0.490
Mastectomy	82 (74.5%)	32 (29.1%)	28 (25.5%)	22 (20.0%)	0.278
Axillary dissection	65 (59.1%)	27 (24.5%)	22 (20.0%)	16 (14.5%)	0.195

Legend: ExG, Structured exercise group; PAC, Brief PA counseling; *CG* Control group. Means ± Standard Deviations are presented for quantitative variables; N (%) are presented for qualitative variables. Differences between groups derived from One-way Anovas or Chi-square tests (*p*-values) are presented in the last column, considering a significance level of *p* < 0.0083

### Primary outcomes

Tables 2 and 3 summarize results for our primary outcomes – PA, sedentary behavior, and quality of life.

**Table 2:** Physical activity and sedentary behavior by groups across time and between-group differences at intervention’s end

Variables	Baseline (pre-intervention)	4 month (post-intervention)	Mean difference Baseline to 4 months	Mean difference Groups at 4 months	Group*Time interaction (*p*-value)	Group main effects (*p*-value)	Time main effects (*p*-value)
*Mean* ± *SE (95%CI)*							
**Total physical activity (accelerometer, GT9X, mean minutes per week)**							
Exercise group (1)	2245 ± 99 (2049; 2440)	2372 ± 100 (2174; 2570)	127 ± 94 (−60; 314)	1 vs 2: 109 ± 143 (−240; 457)			
PAC group (2)	2347 ± 102 (2144; 2550)	2263 ± 104 (2057; 2469)	−84 ± 98 (−278; 110)	2 vs 3: −232 ± 153 (−603; 140)	*0.038*	0.928	0.446
Control group (3)	2210 ± 109 (1994; 2426)	2494 ± 110 (2276; 2714)	**285 ± 104 (79; 491)**	3 vs 1: 123 ± 152 (−248; 493)			
**Moderate-vigorous physical activity (accelerometer, GT9X, mean minutes per week)**							
Exercise group (1)	753 ± 58 (638; 868)	859 ± 61 (739; 980)	*107* ± *52 (4; 209)*	1 vs 2: 137 ± 87 (−75; 349)			
PAC group (2)	781 ± 60 (662; 901)	722 ± 63 (597; 848)	−59 ± 54 (−165; 47)	2 vs 3: −155 ± 93 (−382; 71)	**0.008**	0.775	0.437
Control group (3)	694 ± 64 (567; 821)	877 ± 67 (744; 1011)	**183 ± 57 (70; 296)**	3 vs 1: 18 ± 93 (−208; 244)			
**Light physical activity (accelerometer, GT9X, mean minutes per week)**							
Exercise group (1)	1492 ± 58 (1376; 1608)	1513 ± 52 (1409; 1616)	21 ± 55 (−89; 131)	1 vs 2: −28 ± 75 (−210; 153)			
PAC group (2)	1566 ± 61 (1445; 1686)	1541 ± 54 (1434; 1648)	−25 ± 58 (−139; 89)	2 vs 3: −77 ± 80 (−270; 117)	0.326	0.634	0.568
Control group (3)	1515 ± 65 (1387; 1643)	1617 ± 57 (1503; 1731)	102 ± 61 (−19; 223)	3 vs 1: 105 ± 79 (−88; 298)			
**Sedentary behavior (accelerometer, GT9X, mean hours per day)**							
Exercise group (1)	11.9 ± 0.2 (11.4; 12.3)	12.0 ± 0.3 (11.4; 12.6)	0.1 ± 0.2 (−0.3; 0.6)	1 vs 2: 0.1 ± 0.4 (−0.9; 1.1)			
PAC group (2)	11.7 ± 0.3 (11.1; 12.2)	11.9 ± 0.3 (11.3; 12.5)	0.2 ± 0.2 (−0.2; 0.7)	2 vs 3: 0.2 ± 0.4 (−0.9; 1.3)	0.110	0.878	0.385
Control group (3)	12.1 ± 0.3 (11.6; 12.7)	11.7 ± 0.3 (11.0; 12.3)	−0.5 ± 0.2 (−0.9; 0.03)	3 vs 1: −0.9 ± 24.8 (−1.4; 0.7)			
**Total physical activity (self-reported, IPAQ, mean minutes per week)**							
Exercise group (1)	329 ± 48 (237; 424)	549 ± 60 (430; 669)	**221 ± 67 (88; 353)**	1 vs 2: −3 ± 86 (−213; 208)			
PAC group (2)	373 ± 50 (275; 471)	552 ± 63 (428; 676)	*179* ± *69 (41; 316)*	2 vs 3: 102 ± 92 (−122; 327)	0.414	0.692	0.259
Control group (3)	363 ± 53 (258; 467)	449 ± 67 (317; 582)	87 ± 74 (−59; 233)	3 vs 1: −100 ± 92 (−324; 124)			
**Moderate-vigorous physical activity (self-reported, IPAQ, mean minutes per week)**							
Exercise group (1)	180 ± 34 (110; 249)	316 ± 48 (222; 410)	*136* ± *58 (21; 252)*	1 vs 2: −0.4 ± 68 (−166; 166)			
PAC group (2)	232 ± 36 (160; 304)	316 ± 49 (218; 414)	84 ± 60 (−36; 204)	2 vs 3: 59 ± 73 (−118; 236)	0.603	0.656	0.505
Control group (3)	209 ± 39 (133; 286)	257 ± 52 (153; 361)	48 ± 64 (−80; 175)	3 vs 1: −59 ± 73 (−235; 118)			
**Light physical activity (self-reported, IPAQ, mean minutes per week)**							
Exercise group (1)	132 ± 21 (91; 174)	248 ± 30 (187; 308)	**115 ± 28 (60; 170)**	1 vs 2: 35 ± 44 (−72; 141)			
PAC group (2)	120 ± 22 (77; 163)	213 ± 32 (150; 276)	**93 ± 29 (36; 150)**	2 vs 3: 21 ± 47 (−92; 135)	0.199	0.743	0.167
Control group (3)	152 ± 23 (106; 198)	192 ± 34 (125; 259)	40 ± 31 (−21; 101)	3 vs 1: 19 ± 32 (−58; 97)			
**Sedentary behavior (self-reported, IPAQ, mean hours per day)**							
Exercise group (1)	4.9 ± 0.4 (4.2; 5.6)	4.7 ± 0.3 (4.1; 5.3)	−0.1 ± 0.4 (−0.8; 0.6)	1 vs 2:−0.2 ± 0.4 (−1.3; 0.8)			
PAC group (2)	5.5 ± 0.4 (4.7; 6.2)	5.0 ± 0.3 (4.4; 5.8)	−0.5 ± 0.4 (−1.2; 0.2)	2 vs 3: 0.9 ± 0.5 (−0.2; 2.0)	0.359	0.208	*0.012*
Control group (3)	4.9 ± 0.4 (4.2; 5.7)	4.0 ± 0.3 (3.4; 4.7)	*−0.9* ± *0.4 (−1.7; −0.1)*	3 vs 1: −0.7 ± 0.4 (−1.8; 0.4)			
**Lifestyle physical activity (ACI, 6–30)**							
Exercise group (1)	17.8 ± 0.8 (16.2; 19.3)	19.9 ± 0.7 (18.5; 21.3)	**2.2 ± 0.8 (0.6; 3.7)**	1 vs 2:−0.5 ± 1.0 (−2.5; 2.4)			
PAC group (2)	17.3 ± 0.8 (15.7; 18.9)	20.0 ± 0.7 (18.5; 21.4)	**2.7 ± 0.8 (1.0; 4.3)**	2 vs 3: 1.4 ± 1.1 (−1.2; 4.0)	0.903	0.259	0.564
Control group (3)	16.1 ± 0.9 (14.4; 17.9)	18.6 ± 0.8 (17.0; 20.1)	**2.4 ± 0.9 (0.7; 4.2)**	3 vs 1: −1.3 ± 1.1 (−4.0; 1.3)			

Legend: Mean ± SE (95%CI), Mean, standard error and 95% confidence interval

*IPAQ* International Physical Activity Questionnaire, *ACI* Activity Choice Index

Bold highlights represent significant differences/effects (*p* < 0.008); Italic highlights represent near significant differences/effects (*p* < 0.05)

**Table 3:** Quality of life scores by groups across time and between-group differences at intervention’s end

Variables	Baseline (pre-intervention)	4 month (post-intervention)	Mean difference Baseline to 4 months	Mean difference Groups at 4 months	Group*Time interaction (*p*-value)	Group main effects (*p*-value)	Time main effects (*p*-value)
*Mean* ± *SE (95%CI)*							
**Global health status (EORTC QLQ-C30, 0–100)**							
Exercise group (1)	62.8 ± 3.0 (56.8; 68.8)	69.9 ± 2.4 (65.1; 74.7)	**7.1 ± 2.6 (2.0; 12.2)**	1 vs 2: 3.5 ± 3.4 (−4.9; 11.9)			
PAC group (2)	62.6 ± 3.1 (56.4; 68.8)	66.4 ± 2.5 (61.5; 71.4)	3.8 ± 2.7 (−1.5; 9.1)	2 vs 3: −0.8 ± 3.7 (−9.7; 8.2)	0.640	0.775	0.328
Control group (3)	60.6 ± 3.3 (54.0; 67.2)	67.2 ± 2.7 (61.9; 72.4)	*6.6* ± *2.8 (0.9; 12.2)*	3 vs 1: −2.7 ± 3.7 (−11.6; 6.2)			
**Physical functioning (EORTC QLQ-C30, 0–100)**							
Exercise group (1)	87.4 ± 2.0 (83.4; 91.3)	89.4 ± 1.9 (85.7; 93.1)	2.0 ± 1.7 (−1.3; 5.3)	1 vs 2: −4.9 ± 2.7 (−1.6; 11.4)			
PAC group (2)	82.8 ± 2.1 (78.7; 86.9)	84.5 ± 1.9 (80.7; 88.3)	1.7 ± 1.7 (−1.7; 5.1)	2 vs 3: 4.9 ± 2.8 (−2.1; 11.8)	0.056	*0.028*	0.829
Control group (3)	83.2 ± 2.2 (78.9; 87.6)	79.6 ± 2.1 (75.6; 83.7)	−3.6 ± 1.8 (−7.2; 0.04)	**3 vs 1: −9.7 ± 2.8 (−16.6; −2.8)**			
**Role functioning (EORTC QLQ-C30, 0–100)**							
Exercise group (1)	86.4 ± 3.4 (79.6; 93.2)	90.7 ± 2.7 (85.4; 96.0)	4.3 ± 3.5 (−2.7; 11.3)	1 vs 2: 6.9 ± 3.8 (−2.4; 16.3)			
PAC group (2)	79.7 ± 3.6 (72.6; 86.7)	83.7 ± 2.8 (78.2; 89.3)	4.1 ± 3.7 (−3.2; 11.4)	2 vs 3: −2.9 ± 4.1 (−12.9; 7.1)	0.849	0.145	0.828
Control group (3)	79.7 ± 3.8 (72.2; 87.2)	86.6 ± 3.0 (80.7; 92.5)	6.9 ± 3.9 (−0.8; 14.7)	3 vs 1: −4.1 ± 4.1 (−14.0; 5.9)			
**Emotional functioning (EORTC QLQ-C30, 0–100)**							
Exercise group (1)	75.6 ± 3.5 (68.7; 82.5)	72.4 ± 3.0 (66.5; 78.2)	−3.2 ± 3.7 (−10.6; 4.1)	1 vs 2: −2.7 ± 4.2 (−13.0; 7.7)			
PAC group (2)	69.7 ± 3.6 (62.5; 76.8)	75.0 ± 3.1(68.9; 81.1)	5.4 ± 3.8 (−13.0; 2.3)	2 vs 3: 3.3 ± 4.5 (−11.6; 10.4)	0.233	0.901	0.762
Control group (3)	73.5 ± 3.8 (65.9; 81.1)	71.7 ± 3.3 (65.2; 78.2)	−1.7 ± 4.1 (−9.8; 6.3)	3 vs 1: −0.6 ± 4.5 (−11.6; 6.2)			
**Cognitive functioning (EORTC QLQ-C30, 0–100)**							
Exercise group (1)	80.4 ± 3.3 (73.9; 87.0)	79.4 ± 3.0 (73.5; 85.3)	−1.1 ± 2.9 (−6.9; 4.8)	1 vs 2: 1.6 ± 4.2 (−8.7; 12.0)			
PAC group (2)	80.0 ± 3.4 (73.1; 86.7)	77.7 ± 3.1 (71.6; 83.8)	−2.0 ± 3.0 (−8.3; 3.8)	2 vs 3: 3.5 ± 4.6 (−12.0; 8.7)	0.867	0.632	0.759
Control group (3)	77.6 ± 3.7 (70.4; 84.9)	74.2 ± 3.3 (67.7; 80.7)	−3.4 ± 3.2 (−9.9; 3.0)	3 vs 1: −5.2 ± 4.5 (−16.2; 5.9)			
**Social functioning (EORTC QLQ-C30, 0–100)**							
Exercise group (1)	87.7 ± 3.5 (80.8; 94.6)	88.2 ± 2.7 (82.8; 93.6)	0.5 ± 3.4 (−6.2; 7.3)	1 vs 2: −1.2 ± 3.9 (−10.8; 8.3)			
PAC group (2)	86.3 ± 3.6 (79.2; 93.5)	89.5 ± 2.8 (83.8; 95.1)	3.1 ± 3.5 (−3.9; 10.1)	2 vs 3: 2.3 ± 4.2 (−7.9; 12.5)	0.544	0.543	0.229
Control group (3)	80.9 ± 3.8 (73.3; 88.5)	87.1 ± 3.0 (81.1; 93.1)	6.3 ± 3.7 (−1.2; 13.7)	3 vs 1: −1.1 ± 4.2 (−11.2; 4.1)			
**Fatigue (EORTC QLQ-C30, 0–100)**							
Exercise group (1)	26.3 ± 3.2 (19.9; 32.6)	23.4 ± 2.7 (17.9; 28.6)	−3.0 ± 3.0 (−9.0; 3.0)	1 vs 2: −5.9 ± 3.9 (−15.3; 3.5)			
PAC group (2)	32.6 ± 3.3 (26.0; 39.2)	29.2 ± 2.8 (23.7; 34.8)	−3.4 ± 3.1 (−9.6; 2.8)	2 vs 3: 3.0 ± 4.1 (−7.0; 13.1)	0.673	0.214	0.722
Control group (3)	25.7 ± 3.5 (18.7; 32.7)	26.2 ± 3.0 (20.3; 32.1)	0.4 ± 3.3 (−6.2; 7.0)	3 vs 1: 2.9 ± 4.1 (−7.1; 12.9)			
**Pain (EORTC QLQ-C30, 0–100)**							
Exercise group (1)	27.0 ± 4.1 (18.8; 35.2)	22.4 ± 4.3(13.9; 31.0)	−4.6 ± 4.5 (−13.5; 4.4)	**1 vs 2: −22.6 ± 6.2 (−37.6; −7.5)**			
PAC group (2)	34.5 ± 4.3 (26.0; 43.0)	45.0 ± 4.5 (36.1; 53.9)	*10.5* ± *4.7 (1.3; 19.8)*	2 vs 3: 11.1 ± 6.6 (−5.0; 27.1)	0.060	*0.015*	0.094
Control group (3)	34.2 ± 4.6 (25.2; 43.2)	33.9 ± 4.8 (24.5; 43.4)	−0.3 ± 5.0 (−10.1; 9.6)	3 vs 1: 11.5 ± 6.6 (−4.5; 27.5)			
**Insomnia (EORTC QLQ-C30, 0–100)**							
Exercise group (1)	38.7 ± 5.2 (28.4; 49.1)	35.0 ± 4.5 (26.1; 43.9)	−3.8 ± 5.4 (−14.5; 6.9)	1 vs 2: −8.0 ± 6.4 (−23.7; 7.6)			
PAC group (2)	38.9 ± 5.4 (28.2; 49.7)	43.0 ± 4.7 (33.7; 52.2)	4.1 ± 5.6 (−7.1; 15.2)	2 vs 3: 12.9 ± 6.9 (−3.8; 29.6)	0.559	0.314	0.646
Control group (3)	32.8 ± 5.8 (21.4; 44.3)	30.1 ± 5.0 (20.3; 39.9)	−2.7 ± 6.0 (−14.6; 9.1)	3 vs 1: −4.8 ± 6.8 (−21.5; 11.8)			
**Financial difficulties (EORTC QLQ-C30, 0–100)**							
Exercise group (1)	11.8 ± 4.4 (3.1; 25.1)	14.4 ± 3.5 (7.5; 21.2)	2.6 ± 3.5 (−4.4; 9.6)	1 vs 2: 2.2 ± 5.0 (−9.8; 14.3)			
PAC group (2)	17.6 ± 4.5 (8.6; 26.7)	12.2 ± 3.6 (5.1; 19.3)	−5.5 ± 3.7 (−12.7; 1.8)	2 vs 3: 3.3 ± 5.3 (−9.5; 16.2)	0.159	0.871	0.292
Control group (3)	15.5 ± 4.8 (5.9; 25.1)	8.8 ± 3.8 (1.3; 16.4)	−6.6 ± 3.9 (−14.3; 1.1)	3 vs 1: −5.5 ± 5.3 (−18.4; 7.3)			
**Body image (EORTC QLQ-BR23, 0–100)**							
Exercise group (1)	74.2 ± 4.2 (65.9; 82.4)	80.6 ± 3.6 (73.4; 87.7)	*6.4* ± *3.2 (0.1; 12.7)*	1 vs 2: −1.0 ± 5.2 (−13.6; 11.6)			
PAC group (2)	67.8 ± 4.3 (59.2; 76.3)	81.6 ± 3.7 (74.1; 89.0)	**13.8 ± 3.3 (7.2; 20.4)**	2 vs 3: 10.2 ± 5.5 (−3.2; 23.7)	0.070	0.390	0.921
Control group (3)	68.5 ± 4.6 (59.4; 77.6)	71.3 ± 4.0 (63.4; 79.2)	2.8 ± 3.5 (−4.2; 9.8)	3 vs 1: −9.2 ± 5.5 (−22.6; 4.2)			
**Sexual functioning (EORTC QLQ-BR23, 0–100)**							
Exercise group (1)	83.3 ± 3.0 (77.4; 89.2)	75.5 ± 2.7 (70.0; 80.9)	*−7.9* ± *3.4 (−14.6; −1.2)*	1 vs 2: −0.1 ± 3.9 (−9.7; 9.4)			
PAC group (2)	79.1 ± 3.1 (73.0; 85.2)	75.6 ± 2.9 (69.9; 81.2)	−3.5 ± 3.5 (−10.5; 3.4)	2 vs 3: −1.5 ± 4.2 (−11.7; 8.7)	0.653	0.673	0.621
Control group (3)	83.7 ± 3.3 (77.2; 90.2)	77.1 ± 3.0 (71.1; 83.1)	−6.6 ± 3.7 (−14.0; 0.8)	3 vs 1: 1.6 ± 4.2 (−8.6; 11.8)			
**Future perspectives (EORTC QLQ-BR23, 0–100)**							
Exercise group (1)	60.1 ± 5.2 (49.8; 68.6)	59.2 ± 4.7 (49.9; 68.6)	−0.8 ± 5.2 (−11.2; 9.6)	1 vs 2:−2.0 ± 6.8 (−18.5; 14.5)			
PAC group (2)	55.9 ± 5.4 (45.2; 66.5)	61.2 ± 4.9 (51.5; 71.0)	5.4 ± 5.4 (−5.4; 16.2)	**2 vs 3: 23.7 ± 7.2 (6.2; 41.3)**	0.128	*0.020*	0.932
Control group (3)	48.5 ± 5.7 (37.2; 59.8)	37.5 ± 5.2 (27.1; 47.8)	−11.0 ± 5.8 (−22.5; 0.5)	*3 vs 1: −21.8* ± *7.2 (−39.3; −4.2)*			
**Systemic therapy side effects (EORTC QLQ-BR23, 0–100)**							
Exercise group (1)	15.1 ± 2.3 (10.5; 19.6)	15.4 ± 1.7 (12.0; 18.8)	0.3 ± 1.8 (−3.3; 3.9)	1 vs 2: −0.01 ± 2.5 (−6.0; 6.0)			
PAC group (2)	18.0 ± 2.4 (13.2; 22.7)	15.4 ± 1.8 (11.9; 18.9)	−2.6 ± 1.9 (−6.3; 1.2)	2 vs 3: −4.2 ± 2.6 (−10.6; 2.1)	0.421	0.340	*0.018*
Control group (3)	18.9 ± 2.5 (13.9; 23.9)	19.6 ± 1.9 (15.9; 23.41)	0.7 ± 2.0 (−3.3; 4.7)	3 vs 1: 4.2 ± 2.6 (−2.1; 10.6)			
**Breast symptoms (EORTC QLQ-BR23, 0–100)**							
Exercise group (1)	18.8 ± 3.3 (12.2; 25.4)	15.2 ± 2.5 (10.1; 20.2)	−3.2 ± 3.3 (−9.7; 3.4)	1 vs 2:−0.2 ± 3.6 (−9.1; 8.7)			
PAC group (2)	27.3 ± 3.5 (20.4; 34.2)	15.4 ± 2.6 (10.1; 20.6)	**−11.9 ± 3.1 (−18.1; −5.8)**	2 vs 3: −1.7 ± 3.9 (−11.1; 7.7)	0.084	0.491	0.962
Control group (3)	20.2 ± 3.7 (12.9; 27.5)	17.1 ± 2.8 (11.5; 22.6)	−3.2 ± 3.3 (−9.7; 3.4)	3 vs 1: 1.9 ± 3.9 (−7.5; 11.3)			
**Arm symptoms (EORTC QLQ-BR23, 0–100)**							
Exercise group (1)	21.4 ± 3.5 (14.6; 28.3)	18.1 ± 2.6 (12.9; 23.3)	−3.3 ± 3.3 (−9.9; 3.2)	1 vs 2:−1.2 ± 3.8 (−10.4; 8.0)			
PAC group (2)	24.9 ± 3.6 (17.8; 32.1)	19.3 ± 2.7 (13.8; 24.7)	−5.7 ± 3.4 (−12.5; 1.1)	2 vs 3: −1.7 ± 4.0 (−11.5; 8.1)	0.192	0.739	*0.040*
Control group (3)	17.7 ± 3.8 (10.1; 25.2)	21.0 ± 2.9 (15.2; 26.7)	3.3 ± 3.6 (−3.9; 10.5)	3 vs 1: 2.9 ± 4.0 (−6.9; 12.7)			
**Musculoskeletal symptoms (EORTC QLQ-BR45, 0–100)**							
Exercise group (1)	32.9 ± 4.3 (24.5; 41.4)	27.5 ± 3.8 (20.1; 35.0)	−5.4 ± 4.2 (−13.8; 3.0)	1 vs 2: −12.7 ± 5.4 (−25.8; 0.4)			
PAC group (2)	45.2 ± 4.4 (36.4; 54.0)	40.2 ± 3.9 (32.5; 48.0)	−4.9 ± 4.4 (−13.7; 3.8)	2 vs 3: 2.6 ± 5.7 (−11.4; 16.6)	0.930	*0.026*	0.134
Control group (3)	44.9 ± 4.7 (35.7; 54.3)	37.6 ± 4.1 (29.4; 45.8)	−7.3 ± 4.7 (−16.6; 2.0)	3 vs 1: 10.1 ± 5.7 (−3.9; 24.0)			

Legend: Mean ± SE (95%CI), Mean, standard error and 95% confidence interval. European Organization for Research and Treatment of Cancer Quality of Life Questionnaire Core 30 (EORTC QLQ-C30) and the breast cancer modules (EORTC QLQ-BR23; EORTC QLQ-BR45). Bold highlights represent significant differences/effects (*p* < 0.008); Italic highlights represent near significant differences/effects (*p* < 0.05)

There were generally no significant main effects of group or time, nor group by time interactions, for objectively measured PA and sedentary behaviors, with one exception. A significant group by time interaction of medium magnitude was found for MVPA (F = 5.11; *p* = 0.008; Eta^2^ = 0.09), with pairwise comparisons showing significant increases in CG from baseline to post-intervention (MD [95% CI]: 183 min/week [70; 296], *p* = 0.002), a near significant increase in ExG (MD [95% CI]: 107 min/week [4; 209], *p* = 0.042) and a change in PAC in the opposite direction (although non-significant). Significant increases in total PA (MD [95% CI]: 285 min/week [79; 491], *p* = 0.007) were also observed in CG from baseline to post-intervention. When considering self-reported PA and sedentary behavior, although we generally did not find significant interaction effects nor main effects of time or group, we did find significant increases in both intervention groups in LPA (MD [95% CI]: ExG – 115 min/week [60; 170], *p* < 0.001; PAC – 93 min/week [36; 150], *p* = 0.002) over time. We also found a significant increase in total PA (MD [95% CI]: 221 min/week [88; 353], *p* = 0.001) and a near significant increase in MVPA (MD [95% CI]: 136 min/week [21; 252], *p* = 0.021) in the exercise group. The increase in total PA observed in PAC approached significance (MD [95% CI]: 179 min/week [41; 316], *p* = 0.011). Also, significant increases in physically active choices over time in all groups (MD [95% CI]: ExG – 2.2 [0.6; 3.7], p|= 0.008; PAC – 2.7 [1.0; 4.3], *p* = 0.002; CG – 2.4 [0.7; 4.2], *p* = 0.007, scale range 6–24). There were no significant differences on self-reported sedentary behavior.

Generally, there were no significant interaction effects, nor main effects of group or time in quality-of-life measures. Pairwise comparisons showed significantly higher scores in physical functioning in the ExG, compared to CG, post-intervention (MD [95% CI]: 9.7 [2.8; 16.6], *p* = 0.003, scale range 0–100). Pairwise comparisons also revealed significant differences in the pain symptoms’ scale between ExG and PAC post-intervention, with lower scores in the former (MD [95% CI]: −22.6 [−37.6; −7.5], *p* = 0.001, scale range 0–100), and a deterioration trend in pain symptoms in PAC over time (MD [95% CI]: 10.5 [1.3; 19.8], *p* = 0.026, scale range 0–100). Future perspectives were significantly different between PAC and CG post-intervention (MD [95% CI]: 23.7 [6.2; 41.3], *p* = 0.004, scale range 0–100) and borderline significant between ExG and CG (MD [95% CI]: 21.8 [4.2; 39.3], *p* = 0.009, scale range 0–100). Additionally, pairwise comparisons showed significant improvements in global health status in the ExG (MD [95% CI]: 7.1 [2.0; 12.2], *p* = 0.007, scale range 0–100) from baseline to post-intervention. Significant improvements in the body image functional scale were observed in PAC (MD [95% CI]: 13.8 [7.2; 20.4], *p* < 0.001, scale range 0–100). A significant improvement in breast symptoms was also observed in the PAC group over time (MD [95% CI]: −11.9 [−18.1; −5.8], *p* < 0.001, scale range 0–100).

### Secondary outcomes

Tables 4 and 5 summarize results for our secondary psychological and physical outcomes.

**Table 4:** Psychological outcomes by groups across time and between-group differences at intervention’s end

Variables	Baseline (pre-intervention)	4 month (post-intervention)	Mean difference Baseline to 4 months	Mean difference Groups at 4 months	Group*Time interaction (*p*-value)	Group main effects (*p*-value)	Time main effects (*p*-value)
*Mean* ± *SE (95%CI)*							
**Life satisfaction (1 item, 1–10)**							
Exercise group (1)	7.1 ± 0.3 (6.5; 7.8)	7.6 ± 0.3 (7.0; 8.2)	0.4 ± 0.3 (−0.2; 1.1)	1 vs 2: −0.1 ± 0.4 (−1.1; 0.9)			
PAC group (2)	6.8 ± 0.3 (6.1; 7.5)	7.7 ± 0.3 (7.1; 8.3)	*0.8* ± *0.3 (0.2; 1.5)*	2 vs 3: 0.6 ± 0.4 (−0.5; 1.7)	0.274	0.729	0.741
Control group (3)	7.0 ± 0.4 (6.3; 7.7)	7.1 ± 0.3 (6.4; 7.7)	0.1 ± 0.3 (−0.6; 0.8)	3 vs 1: −0.5 ± 0.4 (−1.7; 0.5)			
**Life’s worth (1 item, 1–10)**							
Exercise group (1)	7.6 ± 0.3 (7.0; 8.2)	7.9 ± 0.3 (7.3; 8.4)	0.3 ± 0.3 (−0.4; 1.0)	1 vs 2: −0.0 ± 0.4 (−1.0; 1.0)			
PAC group (2)	7.6 ± 0.3 (7.0; 8.2)	7.9 ± 0.3 (7.3; 8.4)	0.3 ± 0.4 (−0.4; 1.0)	2 vs 3: −0.4 ± 0.4 (−1.4; 0.6)	0.993	0.522	0.517
Control group (3)	7.9 ± 0.3 (7.3; 8.6)	8.2 ± 0.3 (7.6; 8.8)	0.3 ± 0.4 (−0.4; 1.1)	3 vs 1: 0.4 ± 0.4 (−0.6; 1.4)			
**Life’s purpose (1 item, 1–10)**							
Exercise group (1)	7.9 ± 0.3 (7.4; 8.5)	8.1 ± 0.2 (7.6; 8.6)	0.1 ± 0.3 (−0.5; 0.8)	1 vs 2: −0.5 ± 0.4 (−1.4; 0.4)			
PAC group (2)	8.0 ± 0.3 (7.4; 8.5)	8.6 ± 0.3 (8.1; 9.1)	*0.6* ± *0.3 (−0.3; 1.3)*	2 vs 3: 0.2 ± 0.4 (−0.7; 1.1)	0.506	0.609	0.075
Control group (3)	8.2 ± 0.3 (7.6; 8.8)	8.4 ± 0.3 (7.8; 8.9)	0.2 ± 0.3 (−0.5; 0.9)	3 vs 1: 0.3 ± 0.4 (−0.6; 1.2)			
**Positive life expectations (1 item, 1–10)**							
Exercise group (1)	9.0 ± 0.2 (8.6; 9.4)	8.5 ± 0.3 (8.0; 9.0)	*−0.5* ± *0.3 (−1.0; 0.5)*	1 vs 2: −0.3 ± 0.4 (−1.2; 0.6)			
PAC group (2)	8.9 ± 0.2 (8.5; 9.4)	8.8 ± 0.3 (8.3; 9.3)	−0.1 ± 0.3 (−0.6; 0.4)	2 vs 3: 0.1 ± 0.4 (−0.8; 1.1)	0.148	0.482	0.361
Control group (3)	8.4 ± 0.2 (7.9; 8.8)	8.7 ± 0.3 (8.1; 9.2)	0.3 ± 0.3 (−0.3; 0.9)	3 vs 1: 0.1 ± 0.4 (−0.8; 1.1)			
**Depression (HADS, 0–21)**							
Exercise group (1)	9.8 ± 0.5 (8.8; 10.9)	10.0 ± 0.5 (89.0; 10.9)	0.2 ± 0.5 (−0.9; 1.2)	1 vs 2: 1.1 ± 0.7(−0.6; 2.7)			
PAC group (2)	10.3 ± 0.5 (9.2; 11.4)	8.9 ± 0.5 (7.9; 9.9)	*−1.4* ± *0.5 (−2.4; −0.3)*	2 vs 3: −0.6 ± 0.7 (−2.4; 1.2)	0.065	0.742	0.576
Control group (3)	9.3 ± 0.6 (8.2; 10.5)	9.5 ± 0.5 (8.4; 10.5)	0.2 ± 0.6 (−1.0; 1.3)	3 vs 1: −0.5 ± 0.7 (−2.3; 1.3)			
**Body dissatisfaction (BIS, 0–30)**							
Exercise group (1)	7.7 ± 1.1 (5.4; 9.9)	6.1 ± 1.0 (4.1; 8.2)	*−1.5* ± *0.8 (−3.2; 0.1)*	1 vs 2: 0.7 ± 1.5 (−2.9; 4.4)			
PAC group (2)	9.1 ± 1.2 (6.8; 11.5)	5.4 ± 1.1 (3.3; 7.5)	**−3.8 ± 0.9 (−5.5; −2.0)**	*2 vs 3: −3.5* ± *1.6 (−7.4; 0.4)*	*0.016*	0.390	0.350
Control group (3)	8.9 ± 1.3 (6.4; 11.4)	8.9 ± 1.1 (6.6; 11.2)	−0.4 ± 0.9 (−1.9; 1.8)	3 vs 1: 2.8 ± 1.6 (−1.1; 6.6)			
**Pain average (BPI, 0–10)**							
Exercise group (1)	3.8 ± 0.4 (3.1; 4.6)	3.6 ± 0.3 (2.9; 4.3)	−0.3 ± 0.3 (−0.9; 0.4)	**1 vs 2: −1.5 ± 0.5 (−2.7; −0.4)**			
PAC group (2)	4.5 ± 0.4 (3.8; 5.3)	5.1 ± 0.3 (4.4; 5.8)	*0.6* ± *0.3 (−0.0; 1.2)*	2 vs 3: 0.5 ± 0.5 (−0.7; 1.8)	0.135	0.051	0.330
Control group (3)	4.2 ± 0.4 (3.4; 5.0)	4.6 ± 0.4 (3.9; 5.3)	0.4 ± 0.3 (−0.3; 1.1)	3 vs 1: 1.0 ± 0.5 (−0.2; 2.3)			
**Pain now (BPI, 0–10)**							
Exercise group (1)	2.8 ± 0.4 (2.1; 3.6)	2.8 ± 0.4 (2.1; 3.5)	−0.0 ± 0.4 (−0.8; 0.7)	1 vs 2: −1.1 ± 0.5 (−2.3; 0.1)			
PAC group (2)	2.7 ± 0.4 (1.9; 3.5)	3.9 ± 0.4 (3.2; 4.6)	**1.2 ± 0.4 (0.4; 2.0)**	2 vs 3: 0.7 ± 0.5(−0.6; 2.1)	0.055	0.542	0.123
Control group (3)	3.2 ± 0.4 (2.3; 4.1)	3.1 ± 0.4 (2.4; 3.9)	−0.1 ± 0.4 (−0.9; 0.8)	3 vs 1: 0.3 ± 0.5 (−1.0; 1.7)			
**Pain interference (PDI, 0–70)**							
Exercise group (1)	13.5 ± 1.9 (9.8; 17.1)	12.0 ± 1.8 (8.4; 15.7)	−1.4 ± 1.7 (−4.9; 2.0)	1 vs 2: −4.6 ± 2.6 (−11.0; 1.8)			
PAC group (2)	17.9 ± 1.9 (14.1; 21.7)	16.6 ± 1.9 (12.9; 20.4)	−1.2 ± 1.8 (−4.8; 2.3)	2 vs 3: 2.3 ± 2.8 (−4.5; 9.2)	0.926	0.157	0.742
Control group (3)	16.5 ± 2.0 (12.5; 20.6)	14.3 ± 2.0 (10.3; 18.3)	−2.2 ± 1.9 (−6.0; 1.5)	3 vs 1: 2.3 ± 2.8 (−4.5; 9.1)			
**Sleep quality (PSQI, out of 100)**							
Exercise group (1)	14.1 ± 0.7 (12.7; 15.4)	13.2 ± 0.7 (11.9; 14.6)	−0.8 ± 0.5 (−1.8; 0.2)	1 vs 2: −0.6 ± 1.0 (−3.0; 1.7)			
PAC group (2)	14.4 ± 0.7 (13.0; 15.7)	13.9 ± 0.7 (12.5; 15.2)	−0.5 ± 0.5 (−1.5; 0.6)	2 vs 3: 0.04 ± 1.0 (−2.4; 2.5)	0.776	0.862	**0.005**
Control group (3)	14.1 ± 0.7 (12.6; 15.6)	13.8 ± 0.7 (12.4; 15.3)	−0.3 ± 0.6 (−1.4; 0.8)	3 vs 1: −0.6 ± 1.0 (−1.9; 3.1)			
**Exercise need satisfaction (BPNSFS, 12–60)**							
Exercise group (1)	47.4 ± 1.1 (45.1; 49.6)	50.5 ± 1.1 (48.3; 52.7)	**3.1 ± 1.0 (1.2; 5.1)**	1 vs 2: 3.1 ± 1.6 (−0.7; 7.0)			
PAC group (2)	48.4 ± 1.2 (46.0; 50.8)	47.3 ± 1.1 (45.1; 49.6)	−1.1 ± 1.0 (−3.1; 1.0)	2 vs 3: −1.7 ± 1.7 (−5.8; 2.4)	*0.013*	0.708	0.313
Control group (3)	48.9 ± 1.2 (46.4; 51.4)	49.0 ± 1.2 (46.6; 51.5)	0.2 ± 1.1 (−2.0; 2.3)	3 vs 1: −1.5 ± 1.2 (−5.6; 2.6)			
**Exercise need frustration (BPNSFS, 12–60)**							
Exercise group (1)	24.2 ± 1.2 (21.8; 26.6)	21.6 ± 0.9 (19.9; 23.4)	*−2.6* ± *1.0 (−4.5; −0.7)*	1 vs 2: 0.0 ± 1.3 (−3.0; 3.1)			
PAC group (2)	24.7 ± 1.2 (22.2; 27.1)	21.6 ± 0.9 (19.8; 23.4)	**−3.1 ± 1.0 (−5.1; −1.1)**	2 vs 3: −1.9 ± 1.3 (−5.1; 1.4)	0.427	0.715	0.076
Control group (3)	24.6 ± 1.3 (22.0; 27.2)	23.4 ± 1.0 (21.5; 25.4)	−1.2 ± 1.1 (−3.3; 1.0)	3 vs 1: 1.8 ± 1.3 (−1.4; 5.1)			
**Exercise autonomous motivation (BREQ-3, 0–36)**							
Exercise group (1)	24.7 ± 1.0 (22.6; 26.7)	29.4 ± 1.0 (27.5; 31.3)	**4.7 ± 0.8 (3.2; 6.3)**	1 vs 2: 1.0 ± 1.4 (−2.4; 4.4)			
PAC group (2)	26.8 ± 1.1 (24.6; 28.9)	28.4 ± 1.0 (26.4; 30.4)	*1.6* ± *0.8 (0.0; 3.2)*	2 vs 3: 0.7 ± 1.5 (−2.9; 4.3)	**< 0.001**	0.469	0.242
Control group (3)	29.8 ± 1.1 (27.5; 32.1)	27.7 ± 1.1 (25.6; 29.8)	*−2.1* ± *0.9 (−3.8; −0.4)*	3 vs 1: −1.7 ± 1.5 (−5.3; 1.9)			
**Exercise introjected motivation (BREQ-3, 0–12)**							
Exercise group (1)	4.4 ± 0.5 (3.4; 5.4)	4.9 ± 0.4 (4.1; 5.8)	0.5 ± 0.5 (−0.4; 1.4)	1 vs 2: 0.5 ± 0.6 (−1.1; 2.0)			
PAC group (2)	4.7 ± 0.5 (3.7; 5.7)	4.5 ± 0.5 (3.5; 5.4)	−0.3 ± 0.5 (−1.2; 0.7)	2 vs 3: −0.3 ± 0.7 (−2.0; 1.3)	**0.008**	0.202	0.290
Control group (3)	6.4 ± 0.5 (5.4; 7.5)	4.8 ± 0.5 (3.8; 5.7)	**−1.7 ± 0.5 (−2.7; −0.7)**	3 vs 1: −0.2 ± 0.7 (−1.8; 1.5)			
**Exercise external motivation (BREQ-3, 0–12)**							
Exercise group (1)	1.4 ± 0.3 (0.8; 2.1)	0.7 ± 0.2 (0.3; 1.2)	*−0.7* ± *0.3 (−1.4; −0.1)*	1 vs 2: 0.2 ± 0.3 (−0.6; 1.0)			
PAC group (2)	1.5 ± 0.3 (0.8; 2.1)	0.5 ± 0.2 (3.9; 6.1)	*−0.9* ± *0.3 (−1.6; −0.2)*	2 vs 3: −0.7 ± 0.3 (−1.6; 0.1)	*0.050*	0.929	0.508
Control group (3)	1.0 ± 0.4 (0.3; 1.7)	1.3 ± 0.3 (0.1; 1.0)	0.3 ± 0.4 (−0.5; 1.0)	3 vs 1: 0.5 ± 0.3 (−0.3; 1.4)			
**Exercise amotivation (BREQ-3, 0–12)**							
Exercise group (1)	0.9 ± 0.3 (0.3; 1.4)	0.4 ± 0.2 (0.1; 0.7)	*−0.5* ± *0.3 (−1.1; 0.1)*	1 vs 2:- 0.3 ± 0.2 (−0.9; 0.3)			
PAC group (2)	1.2 ± 0.3 (0.6; 1.7)	0.6 ± 0.2 (0.3; 1.0)	*−0.5* ± *0.3 (−1.1; 0.1)*	2 vs 3: 0.3 ± 0.3 (−0.3; 1.0)	0.352	*0.046*	0.364
Control group (3)	0.2 ± 0.3 (−0.3; 0.8)	0.3 ± 0.2 (−0.1; 0.7)	0.1 ± 0.3 (−0.6; 0.7)	3 vs 1: −0.1 ± 0.3 (−0.7; 0.5)			
**Exercise self-efficacy (Bandura’s ESES, 9–45)**							
Exercise group (1)	22.7 ± 0.8 (21.1; 24.4)	21.3 ± 20.8 (19.7; 22.8)	*−1.5* ± *0.8 (−3.1; 0.1)*	1 vs 2: 0.8 ± 1.1 (−1.9; 3.5)			
PAC group (2)	21–5 ± 0.9 (19.8; 23.2)	20.4 ± 20.8 (18.8; 22.1)	−1.0 ± 0.8 (−2.7; 0.7)	2 vs 3: 1.8 ± 1.2 (−1.7; 4.1)	0.370	*0.028*	0.517
Control group (3)	19.0 ± 0.9 (17.2; 20.8)	19.3 ± 0.9 (17.6; 21.0)	0.2 ± 0.9 (−1.5; 2.0)	3 vs 1: −2.0 ± 1.2 (−4.9; 0.9)			
**Affective Response to exercise (FS, −5 to 5)**							
Exercise group (1)	3.2 ± 0.2 (2.9; 4.0)	4.2 ± 0.2 (3.8; 4.6)	**1.0 ± 0.2 (0.6; 1.5)**	1 vs 2: 0.6 ± 0.3 (−0.1; 1.2)			
PAC group (2)	3.1 ± 0.3 (2.6; 3.6)	3.7 ± 0.2 (3.3; 4.1)	*0.6* ± *0.2 (0.1; 1.0)*	2 vs 3: 0.4 ± 0.3 (−0.3; 1.1)	**0.002**	0.406	0.122
Control group (3)	3.5 ± 0.3 (2.9; 4.0)	3.3 ± 0.2 (2.8; 3.7)	−0.2 ± 0.2 (−0.7; 0.3)	**3 vs 1: −1.0 ± 0.3 (−1.7; −0.3)**			

Legend: Mean ± SE (95%CI), Mean, standard error and 95% confidence interval. HADS, Hospital Anxiety and Depression Scale. BIS, Body Image Scale. BPI, Brief Pain Inventory. PDI, Pain disability Index. PSQI, Pittsburgh Sleep Quality Inventory. BPNSFS, Basic Psychological Need Satisfaction and Frustration Scale. BREQ-3, Behavioral Regulation for Exercise Questionnaire—3. ESES, Bandura’s Exercise Self-Efficacy Scale. FS, Feeling Scale. Bold highlights represent significant differences/effects (*p* < 0.008); Italic highlights represent near significant differences/effects (*p* < 0.05)

**Table 5:** Physical outcomes by groups across time and between-group differences at intervention’s end

Variables		Baseline (pre-intervention)		4 month (post-intervention)	Mean difference Baseline to 4 months	Mean difference Groups at 4 months	Group*Time interaction (*p*-value)	Group main effects (*p*-value)	Time main effects (*p*-value)
*Mean* ± *SE (95%CI)*									
**Body mass index (kg/m**^**2**^**)**									
Exercise group (1)		28.2 ± 0.7 (26.7; 29.7)		28.4 ± 0.8 (26.7; 29.7)	0.2 ± 0.2 (−0.1; 0.6)	1 vs 2: 0.3 ± 1.1 (−2.4; 2.9)			
PAC group (2)		28.1 ± 0.8 (26.6; 29.7)		28.2 ± 0.8 (26.6; 29.7)	0.1 ± 0.2 (−0.3; 0.4)	2 vs 3: 1.5 ± 1.1 (−1.3; 4.2)	0.461	0.343	*0.038*
Control group (3)		26.8 ± 0.8 (25.2; 28.5)		26.7 ± 0.8 (25.1; 28.4)	−0.1 ± 0.2 (−0.5; 0.3)	3 vs 1: −1.7 ± 1.1 (−4.5; 1.1)			
**Waist circumference (cm)**									
Exercise group (1)		91.3 ± 1.8 (87.8; 94.8)		89.4 ± 1.6 (86.1; 92.6)	**−1.9 ± 0.7 (−3.3; −0.5)**	1 vs 2: 0.4 ± 2.3 (−5.3; 6.1)			
PAC group (2)		91.2 ± 1.8 (87.5; 94.9)		89.0 ± 1.7 (85.6; 92.4)	**−2.2 ± 0.7 (−3.7; −0.7)**	2 vs 3: 3.3 ± 2.5 (−2.8; 9.3)	0.307	0.175	0.384
Control group (3)		86.3 ± 2.0 (82.4; 90.2)		85.7 ± 1.8 (82.2; 89.3)	−0.6 ± 0.8 (−2.2; 0.9)	3 vs 1: −3.7 ± 2.5 (−9.7; 2.3)			
**Body fat (BIA, %)**									
Exercise group (1)		36.4 ± 0.8 (34.7; 38.0)		35.0 ± 0.8 (33.4; 36.7)	**−1.3 ± 0.5 (−2.3; −0.4)**	1 vs 2: −1.3 ± 1.2 (−4.1; 1.6)			
PAC group (2)		37.2 ± 0.9 (35.5; 38.9)		36.3 ± 0.8 (34.6; 38.0)	*−0.9* ± *0.5 (−1.9; 0.1)*	2 vs 3: 1.1 ± 1.2 (−1.9; 4.1)	0.702	0.544	0.288
Control group (3)		36.0 ± 0.9 (34.2; 37.8)		35.2 ± 0.9 (33.4; 37.0)	−0.8 ± 0.5 (−1.8; 0.3)	3 vs 1: 0.2 ± 1.2 (−2.8; 3.2)			
**Cardiorespiratory fitness (VO**_**2**_**max, ml.kg-1.min-1)**									
Exercise group (1)		30.0 ± 0.7 (28.6; 31.3)		32.4 ± 0.5 (31.4; 33.5)	**2.5 ± 0.6 (1.3; 3.7)**	*1 vs 2: 2.1* ± *0.8 (0.3; 4.0)*			
PAC group (2)		29.1 ± 0.7 (27.7; 30.5)		30.3 ± 0.6 (29.2; 31.4)	1.2 ± 0.6 (−0.0; 2.4)	2 vs 3: −0.5 ± 0.8 (−2.5; 1.5)	0.078	0.138	0.944
Control group (3)		30.3 ± 0.7 (28.9; 31.8)		30.8 ± 0.6 (29.6; 31.9)	0.4 ± 0.7 (−0.9; 1.7)	3 vs 1: −1.7 ± 0.8 (−3.7; 0.3)			
**Handgrip strength – best limb (kg)**									
Exercise group (1)		24.0 ± 0.7 (22.6; 25.4)		23.7 ± 0.7 (22.3; 25.1)	−0.3 ± 0.5 (−1.3; 0.7)	1 vs 2: 1.1 ± 1.0 (−1.4; 3.6)			
PAC group (2)		22.7 ± 0.7 (21.2; 24.1)		22.6 ± 0.7 (21.1; 24.1)	−0.1 ± 0.5 (−1.1; 0.9)	2 vs 3: −2.2 ± 1.1 (−4.9; 0.4)	0.907	0.103	0.474
Control group (3)		24.8 ± 0.8 (23.3; 26.3)		24.8 ± 0.8 (23.2; 26.4)	0.02 ± 0.5 (−1.1; 1.1)	3 vs 1: 1.1 ± 1.1 (−1.6; 3.7)			
**Handgrip strength – worst limb (kg)**									
Exercise group (1)		20.4 ± 0.9 (18.6; 22.3)		23.1 ± 0.7 (21.8; 24.5)	**2.7 ± 0.8 (1.1; 4.3)**	*1 vs 2: 2.8* ± *1.0 (0.4; 5.2)*			
PAC group (2)		19.1 ± 1.0 (17.2; 21.1)		20.3 ± 0.7 (18.9; 21.7)	1.2 ± 0.8 (−0.5; 2.8)	*2 vs 3: −3.1* ± *1.0 (−5.6; −0.5)*	0.267	0.075	0.068
Control group (3)		20.4 ± 1.0 (18.3; 22.5)		23.4 ± 0.8 (21.9; 24.9)	**3.0 ± 0.9 (1.2; 4.7)**	3 vs 1: 0.2 ± 1.0 (−2.3; 2.8)			
**Maximal front upper body strength (Chest Press, 10-RM, kg)**									
Exercise group (1)		12.1 ± 0.7 (10.7; 13.3)		16.2 ± 0.7 (14.9; 17.6)	**4.2 ± 0.7 (2.7; 5.6)**	*1 vs 2: 2.9* ± *1.0 (0.5; 5.2)*			
PAC group (2)		11.7 ± 0.7 (10.3; 13.0)		13.4 ± 0.7 (12.0; 14.8)	*1.7* ± *0.8 (0.2; 3.2)*	2 vs 3: 0.1 ± 1.0 (−2.4; 2.6)	*0.021*	0.081	0.062
Control group (3)		11.8 ± 0.7 (10.4; 13.2)		13.3 ± 0.8 (11.8; 14.8)	1.5 ± 0.8 (−0.1; 3.1)	*3 vs 1: −3.0* ± *1.0 (−5.5; −0.4)*			
**Maximal back upper body strength (Seated Row, 10-RM, kg)**									
Exercise group (1)		19.0 ± 0.8 (17.5; 20.6)		23.6 ± 0.6 (22.4; 24.9)	**4.6 ± 0.7 (3.2; 6.0)**	*1 vs 2: 2.3* ± *0.9 (0.1; 4.5)*			
PAC group (2)		18.7 ± 0.8 (17.1; 20.4)		21.3 ± 0.6 (20.0; 22.6)	**2.6 ± 0.7 (1.1; 4.0)**	2 vs 3: 0.3 ± 0.9 (−2.0; 2.6)	**0.006**	0.330	0.600
Control group (3)		19.8 ± 0.9 (18.1; 21.5)		21.0 ± 0.7 (19.6; 22.4)	1.2 ± 0.8 (−0.4; 2.7)	*3 vs 1: −2.6* ± *1.0 (−4.9; −0.3)*			
**Maximal leg strength (Leg Press, 10-RM, kg)**									
Exercise group (1)		78.0 ± 3.8 (70.5; 85.6)		117.6 ± 3.6 (110.5; 124.8)	**39.6 ± 3.3 (33.1; 46.1)**	**1 vs 2: 31.5 ± 5.2 (19.0; 44.1)**			
PAC group (2)		75.9 ± 4.0(68.0; 83.7)		86.1 ± 3.7 (78.7; 93.5)	**10.2 ± 3.4 (3.5; 16.9)**	2 vs 3: 0.2 ± 5.5 (−13.2; 13.5)	** < 0.001**	** < 0.001**	0.712
Control group (3)		77.2 ± 4.2 (68.9; 85.6)		85.9 ± 4.0 (78.0; 93.8)	*8.7* ± *3.6 (1.5; 15.8)*	**3 vs 1: −31.7 ± 5.5 (−45.0;−18.3)**			
**Functional mobility (Timed Up and Go test, seconds)**									
Exercise group (1)		5.0 ± 0.1 (4.8; 5.2)		4.2 ± 0.1 (4.1; 4.4)	**−0.7 ± 0.1 (−1.0; −0.5)**	**1 vs 2: −0.4 ± 0.1 (−0.6; −0.1)**			
PAC group (2)		5.1 ± 0.1 (4.9; 5.3)		4.6 ± 0.1 (4.4; 4.8)	**−0.5 ± 0.1 (−0.7; −0.2)**	2 vs 3: 0.2 ± 0.1 (−0.1; 0.5)	0.143	0.053	0.642
Control group (3)		4.8 ± 0.1 (4.6; 5.1)		4.4 ± 0.1 (4.3; 4.6)	**−0.4 ± 0.1 (−0.7; −0.1)**	3 vs 1: 0.2 ± 0.1 (−0.1; 0.5)			
**Leg strength/endurance (Sit to Stand test, number of sit to stands)**									
Exercise group (1)		15.0 ± 0.6 (13.8; 16.2)		19.4 ± 0.7 (18.1; 20.7)	**4.4 ± 0.6 (3.3; 5.5)**	**1 vs 2: 4.4 ± 0.9 (2.2; 6.7)**			
PAC group (2)		13.3 ± 0.6 (12.1; 14.6)		15.0 ± 0.7 (13.6; 16.3)	**1.7 ± 0.6 (0.5; 2.8)**	*2 vs 3: −3.0* ± *1.0 (−5.5; −0.6)*	**0.002**	**0.001**	0.870
Control group (3)		13.9 ± 0.7 (12.5; 15.2)		18.0 ± 0.7 (16.6; 19.5)	**4.2 ± 0.6 (2.9; 5.4)**	3 vs 1: −1.4 ± 1.0 (−3.8; 1.0)			
**Flexibility – best limb (Back Scratch test, cm)**									
Exercise group (1)		−9.6 ± 1.8 (−13.2; −6.0)		−8.9 ± 1.7 (−12.3; −5.5)	0.7 ± 1.4 (−2.0; 3.4)	1 vs 2: 3.1 ± 2.4 (−2.9; 9.0)			
PAC group (2)		−12.5 ± 1.9 (−16.2; −8.8)		−12.0 ± 1.8 (−15.5; −8.5)	0.5 ± 1.4 (−2.3; 3.4)	2 vs 3: −5.2 ± 2.6 (−11.6; 1.1)	0.992	0.104	0.176
Control group (3)		−7.2 ± 2.0 (−11.1; −3.2)		−6.8 ± 1.9 (−10.4; −3.0)	0.4 ± 1.5 (−2.6; 3.4)	3 vs 1: 2.2 ± 2.6 (−4.2; 8.5)			
**Flexibility – worst limb (Back Scratch test, cm)**									
Exercise group (1)		−17.5 ± 2.1 (−21.6; −13.4)		−13.8 ± 1.5 (−16.9; −10.8)	*3.6* ± *1.4 (0.9; 6.4)*	1 vs 2: 2.6 ± 2.2 (−2.8; 8.0)			
PAC group (2)		−20.2 ± 2.2 (−24.5; −15.9)		−16.4 ± 1.6 (−19.6; −13.3)	*3.7* ± *1.4 (0.9; 6.6)*	2 vs 3: −1.5 ± 2.4 (−7.2; 4.2)	0.299	0.429	0.782
Control group (3)		−15.7 ± 2.3 (−20.3; −11.2)		−14.9 ± 1.7 (−18.3; −11.6)	0.8 ± 1.5 (−2.2; 3.8)	3 vs 1: −1.1 ± 2.4 (−6.8; 4.6)			
**Balance right leg (Stand on one foot test, seconds)**									
Exercise group (1)	15.5 ± 0.9 (13.8; 17.2)		16.5 ± 0.8 (15.0; 18.0)		1.0 ± 1.0 (−0.1; 3.0)	1 vs 2: −0.2 ± 1.1 (−2.9; 2.4)			
PAC group (2)	15.1 ± 0.9 (13.3; 16.9)		16.7 ± 0.8 (15.1; 18.3)		1.6 ± 1.0 (−0.5; 3.7)	2 vs 3: 0.4 ± 1.2 (−2.5; 3.2)	0.897	0.988	0.104
Control group (3)	15.4 ± 1.0 (13.4; 17.3)		16.4 ± 0.8 (14.4; 18.0)		1.0 ± 1.1 (−1.2; 3.2)	3 vs 1: −0.2 ± 1.2 (−3.0; 2.7)			
**Balance left leg (Stand on one foot test, seconds)**									
Exercise group (1)	15.3 ± 0.9 (13.5; 17.0)		15.9 ± 0.7 (14.4; 17.4)		0.6 ± 0.9 (−1.1; 2.3)	1 vs 2: −0.7 ± 1.1 (−3.3; 2.0)			
PAC group (2)	15.4 ± 0.9 (13.6; 17.2)		16.5 ± 0.8 (15.0; 18.1)		1.2 ± 0.9 (−0.6; 2.9)	2 vs 3: 2.3 ± 1.1 (−0.5; 5.0)	0.053	0.797	0.321
Control group (3)	16.2 ± 1.0 (14.3; 18.2)		14.3 ± 0.8 (12.7; 15.9)		**−1.9 ± 0.9 (−3.8; −0.3)**	3 vs 1: −1.6 ± 1.1 (−4.4; 1.2)			
**Sleep duration (accelerometer, GT9X, mean hours per day)**									
Exercise group (1)		6.7 ± 0.1 (6.4; 6.9)		6.4 ± 0.1 (6.1; 6.7)	*−0.3* ± *0.1 (−0.5; −0.2)*	1 vs 2: −0.3 ± 0.2 (−0.8; 0.3)			
PAC group (2)		6.7 ± 0.1 (6.4; 7.0)		6.7 ± 0.2 (6.3; 7.0)	−0.3 ± 0.1 (−0.3; 0.2)	2 vs 3: 0.4 ± 0.2 (−0.1; 1.0)	0.117	0.421	*0.014*
Control group (3)		6.6 ± 0.2 (6.3; 6.9)		6.2 ± 0.2 (5.9; 6.5)	**−0.4 ± 0.1 (−0.7; −0.1)**	3 vs 1: −0.2 ± 0.2 (−0.7; 0.4)			

Legend: Mean ± SE (95%CI), Mean, standard error and 95% confidence interval. BIA, Bioimpedance. VO_2_max, Maximal Oxygen Consumption. Bold highlights represent significant differences/effects (*p* < 0.008); Italic highlights represent near significant differences/effects (*p* < 0.05)

There were no significant group by time interactions, nor main effects of group or time, for subjective well-being indicators. Still, pairwise comparisons showed that improvements in life satisfaction (MD [95% CI]: 0.8 [0.2; 1.5], *p* = 0.011, scale range 1–10) and depression from baseline to post-intervention in the PAC group (MD [95% CI]: −1.4 [−2.4; −0.3], *p* = 0.011, scale range 0–21) approached significance. Pairwise comparisons revealed a significant reduction in body dissatisfaction from baseline to post-intervention in the PAC group (MD [95% CI]: −3.8 [−5.5; −2.0], *p* < 0.001, scale range 0–30), but a significant deterioration in pain scores (MD [95% CI]: 1.2 [0.4; 2.0], *p* = 0.005, scale range 0–10). The ExG showed lower pain scores at post-intervention compared to the PAC group (MD [95% CI]: −1.5 [−2.7; −0.4], *p* = 0.006, scale range 0–10).

Regarding exercise-related psychological outcomes, a significant group by time interaction effect of medium–high magnitude was found for affective response to exercise (F = 6.72; *p* = 0.002; Eta^2^ = 0.12). Pairwise comparisons showed significant improvements in ExG over time (MD [95% CI]: 1.0 [0.6; 1.5], *p* < 0.001, scale range −5 to + 5). As a result, significant differences between ExG and CG were found after the intervention (MD [95% CI]: 1.0 [0.3; 1.7], *p* = 0.005, scale range −5 to + 5). The ExG showed a significant increase over time in need satisfaction (MD [95% CI]: 3.1 [1.2; 5.1], *p* = 0.002, scale range 12–60), which contrasted with a non-significant decrease in PAC. On the other hand, a significant reduction in exercise need frustration over time was observed in the PAC group (MD [95% CI]: −3.1 [−5.1; −1.1], *p* = 0.003, scale range 12–60). Consistent with these findings, significant increases over time in autonomous motivations were found for the ExG (MD [95% CI]: 4.7 [3.2; 6.3], *p* < 0.001, scale range 0–36). There were significant group by time interaction effects of medium magnitude for introjected regulation motivations (F = 5.12; *p* = 0.008; Eta^2^ = 0.09), with pairwise comparisons showing a significant decrease in CG over time (MD [95% CI]: −1.7 [−2.7; −0.7], *p* = 0.001, scale range 0–12). The reduction in external regulation in PAC approached significance (MD [95% CI]: −0.9 [−1.6; −0.2], *p* = 0.009, scale range 0–12).

Although there were no significant interactions nor main effects of group or time, we observed significant improvements in waist circumference in both intervention groups (MD [95% CI]: ExG – −1.9 cm [−3.3; −0.5], *p* = 0.008; PAC – −2.2 cm [−3.7; −0.7], *p* = 0.003) and percent body fat in the ExG (MD [95% CI]: −1.3% [−2.3; −0.4], *p* = 0.007) from baseline to post-intervention.

Regarding fitness-related measures, we found significant high-magnitude group by time interaction effects for maximal lower body strength (F = 26.5; *p* < 0.001; Eta^2^ = 0.34) and medium-size interactions for back upper body strength (F = 5.43; *p* = 0.006; Eta^2^ = 0.10). In general, pairwise comparisons revealed greater improvements in ExG over time (although also significant in PAC), which translated into significantly higher scores in this group for lower body strength (MD [95% CI]: vs PAC – 31.5 kg [19.0; 44.1], *p* < 0.001; vs CG – 31.7 kg [18.3; 45.0], *p* < 0.001) at intervention’s end. Group differences for back upper body strength (MD [95% CI]: ExG vs PAC – 2.3 kg [0.1; 4.5], *p* = 0.033; ExG vs CG – 2.6 kg [0.3; 4.9], *p* = 0.021), front upper body strength (MD [95% CI]: ExG vs PAC – 2.9 kg [0.5; 5.2], *p* = 0.013; ExG vs CG – 3.0 kg [0.4; 5.5], *p* = 0.016), and cardiorespiratory fitness (MD [95% CI]: ExG vs PAC – 2.1 ml.kg-1.min-1 [0.3; 4.0], *p* = 0.020) approached significance. There were significant increases in cardiorespiratory fitness over time in the exercise group (MD [95% CI]: 2.5 ml.kg-1.min-1 [1.3; 3.7], *p* < 0.001). Physical function measures generally showed improvements in all groups from baseline to 4 months. Leg strength/endurance (MD [95% CI]: 4.4 sit to stands [2.2; 6.7], *p* < 0.001) and functional mobility (MD [95% CI]: −0.4 s [−0.6; −0.1], *p* = 0.004) were significantly better in ExG than PAC at intervention’s end. Almost significant improvements were observed in the ExG in upper limb flexibility, namely in the one with worst scores at start (MD [95% CI]: ExG – 3.6 cm [0.9; 6.4], *p* = 0.009). The CG also showed significant improvements in some measures over time, namely in functional mobility (MD [95% CI]: −0.4 s [−0.7; −0.1], *p* = 0.006), leg strength/endurance (MD [95% CI]: 4.2 sit to stands [2.9; 5.4], *p* < 0.001), and almost significant in handgrip strength (MD [95% CI]: 3.0 kg [1.2; 4.7], *p* = 0.009). Significant decreases in sleep duration were also observed in CG from baseline to post-intervention (MD [95% CI]: −0.4 h/day [−0.7; −0.1], *p* = 0.003).

Supplementary analyses with completers generally followed the trends observed in intent-to-treat analyses (data not shown).

## Discussion

This study evaluated the PAC-WOMAN RCT, testing two PA interventions for breast cancer patients on aromatase inhibitors: a supervised exercise program (ExG) versus brief PA counseling (PAC). Primary outcomes (PA, sedentary behavior, and quality of life) and secondary physical and psychosocial outcomes were reassessed immediately after the 4-month intervention. Significant group-by-time interactions were observed for MVPA, exercise autonomous and introjected motivations, affective response to exercise, maximal strength and leg strength/endurance. In addition, both groups revealed significant improvements over time, though with distinct patterns. The ExG group showed increases in total PA, likely derived from the increment in MVPA, while the PAC group exhibited increases in LPA. Quality of life improvements also varied: the ExG improved global health status and physical functioning, whereas PAC enhanced future perspectives, body image functionality, and reduced breast symptoms. Similarly, there were significant improvements over time in physiological indicators (e.g., fitness, body composition) in the ExG, while PAC primarily improved psychosocial indicators (e.g., body dissatisfaction, depressive symptoms, life satisfaction).

No significant group, time, or interaction effects were found for objectively-measured PA indicators in either intervention group, except for increases in MVPA over time in the ExG. Broader improvements were observed in all self-reported PA indicators in the ExG, while PAC revealed gains only in LPA and active lifestyle choices. The absence of more marked differences between groups might be partially explained by the post-intervention assessment timing, which took place right after the intervention had ended (i.e., in the week after), leaving no time for participants to integrate physical activity into their daily routines. In addition, previous studies testing PA promotion interventions for breast cancer survivors have reported significant improvements in objective PA [60, 61], but often among insufficiently active participants (below PA recommended doses). Participants in this trial exhibited high baseline PA levels, potentially limiting room for improvement (i.e., ceiling effects) and contributing only to the maintenance of weekly PA levels. Subgroup analysis of participants below PA recommendations (< 150 min/week) revealed significant improvements in self-reported PA measures in both groups, though objective measures remained non-significant. These findings suggest that baseline PA levels may moderate intervention effectiveness, consistent with prior research showing limited changes among participants already meeting PA guidelines [62]. It is very likely that if less active participants had been recruited, a different panorama had been observed. Restricting eligibility to insufficiently active individuals might enhance intervention efficacy but poses recruitment challenges [63].

The average minutes of PA measured by accelerometer exceeded population-based estimates for cancer survivors [64], possibly due to participant characteristics (e.g., motivation) or device limitations. Wrist-worn accelerometers like the GT9X used herein tend to overestimate PA compared to hip-worn devices [65, 66], precluding comparisons between studies employing different PA measurement methodologies. Additionally, existing cut-point algorithms are for hip-worn accelerometers and not tailored to older or chronic disease populations, potentially misestimating activity types and intensities [67, 68]. Energy expenditure associated with a given metabolic equivalent is ~ 71% lower in older adults compared to younger populations [69].

Unexpectedly, significant improvements in objectively-measured MVPA and total PA were observed in CG, as reported in previous physical activity intervention trials [18, 70]. Inclusion of active participants, reactivity to physical activity monitoring, awareness of being involved in an experimental trial, increased self-awareness of behaviors from measurement, or high participant’s readiness for change at baseline might have prompted changes in physical activity regardless of any intervention [71, 72]. Still, these increases in objectively-measured PA were not accompanied by changes in self-reported measures. Accelerometer reactivity, i.e., modifications in movement behaviors driven by increased self-awareness induced by monitoring devices [73], has been empirically confirmed [74, 75] and might explain this discrepancy. Furthermore, it could lead to the misestimation of movement behaviors in repeated measurements, hindering the detection of significant intervention effects [74].

Evidence supports the effectiveness of PA behavior change interventions in enhancing quality of life post-intervention [50, 63, 64]. Consistent with these findings, PAC significantly improved quality-of-life dimensions (e.g., body image functionality, future perspectives), psychosocial outcomes (e.g., depressive symptoms) and life satisfaction. Grounded in self-determination theory, PAC promoted autonomy, competence, and relatedness while fostering self-regulatory skills. Although less effective at increasing overall PA levels than ExG, PAC demonstrated meaningful psychological benefits relevant to cancer survivorship.

In turn, ExG induced significant quality-of-life improvements, particularly in physical domains such as global health status and physical functioning, corroborating prior research [9]. These benefits likely stem from the substantial fitness gains (e.g., cardiorespiratory fitness, strength) observed in ExG, consistent with prior research linking fitness improvements to enhanced functionality and reduced health impairments [76, 77]. In effect, significant group x time interactions were observed for strength-related outcomes, indicating that the supervised exercise intervention produced meaningful improvements in muscular performance, in line with recent evidence [78]. These findings support the efficacy of structured, supervised resistance training programs, in promoting neuronal and muscular adaptations even in populations with health challenges [79, 80]. Improvements in strength may also contribute indirectly to greater physical function, exercise tolerance, and daily activity engagement, as suggested in oncology and rehabilitation settings [81].

In line with the abovementioned, and consistent with the increment in MVPA, significant group by time interactions were also observed for exercise-related affective and motivational variables, resulting in greater improvements in the ExG. More positive affective responses have been linked to MVPA in the literature [82, 83] and also to motivational variables [84]. In this regard, the ExG showed a significant increase over time in need satisfaction and significant increases over time in autonomous motivations as predicted by SDT [21]. Experiencing success, competence, and positive feedback during exercise can increase need satisfaction (competence, autonomy, relatedness) which, in turn, strengthens autonomous motivation — even in programs not formally following SDT principles [20, 85]. Supervised exercise classes usually enhance structure, feedback, and interpersonal connections, which can facilitate competence and relatedness needs. While SDT emphasizes explicit need-supportive environments, exercisers often extract motivational benefits and satisfaction from their experience and progress, even when settings are neutral or mildly controlling [85].

AI adverse effects often lead to poor adherence or therapy discontinuation, reducing treatment efficacy and increasing mortality risk [5, 86]. In line with prior studies [9, 87], ExG participants experienced stable or reduced pain symptoms compared to increased pain among PAC participants. LPA increases observed in PAC may be insufficient to mitigate AI-induced arthralgia. ExG’s pronounced fitness improvements likely contributed to the observed pain reductions.

The PAC-WOMAN trial shows that two distinct PA promotion interventions yield different but unique benefits for breast cancer survivors on AI, underscoring the practical scalability of the brief PA counseling intervention (PAC) and the substantial physical health gains from structured exercise (ExG). These results suggest that these two types of programs can be complementary. In fact, a previous systematic review and meta-analysis on PA maintenance across cancer types, including an analysis of intervention components and context, suggested that reasonably low-intensity interventions may be sufficient in prompting lasting behavior change (although in motivated, young, well-educated, white populations) [18]. This is a very powerful message for public health stakeholders with potential implications for the sustainability of health systems. Nonetheless, the same review found that more intensive support/interventions are likely to be required for other populations, especially for older people and those with physical limitations. Indeed, the inclusion of a supervised component and frequent contact with participants may further increase intervention effectiveness. Thus, a stepped care approach to behavior change (tailored to participant characteristics) is recommended.

This study is not without limitations. Recruitment was not restricted to sedentary or insufficiently active individuals, potentially introducing ceiling effects that underestimated intervention efficacy. The sample predominantly included participants with higher socioeconomic status and educational attainment, limiting generalizability to lower socioeconomic groups. Findings may not extend to survivors with other breast cancer types, different treatment phases, or other cancers.

Future research should assess the long-term sustainability of behavior change interventions and their associated health benefits, with a focus on understanding how and for whom these outcomes persist. It should also explore ways to integrate structured exercise and counseling programs for cancer survivors into routine healthcare, using implementation frameworks and digital tools for scalable, personalized support. Given that participants who seek PA interventions are relatively young, female, well-educated, and predominantly white, addressing social inequalities in current interventions requires inclusive approaches, such as stepped care models tailored to diverse patient needs and supported by trained staff. Collaboration with healthcare organizations and evaluation of cost, feasibility, and acceptability will be essential for successful adoption and scaling-up.

## Conclusions

The PAC-WOMAN trial tested two different PA promotion interventions – ExG and PAC – yielding unique benefits for breast cancer survivors on AI. ExG generally improved all forms of PA, fitness measures, physical function, and physical quality of life. PAC primarily enhanced LPA and psychological outcomes such as body image functionality and life satisfaction. Pain scores were significantly lower in ExG than PAC at intervention’s end, suggesting structured exercise may be necessary to alleviate pain symptoms in this population. These findings highlight the potential scalability of PAC alongside the robust physical benefits of ExG. Further research is needed to evaluate long-term effectiveness, cost-efficiency, and large-scale implementation potential for both interventions.

## Supplementary Information

Supplementary Material 1

Supplementary Material 2

Supplementary Material 3

## Data Availability

Anonymized trial data are available from the corresponding author upon reasonable request for non-commercial research purposes.

## Abbreviations

ACI: Activity Choice Index
AI: Aromatase inhibitors
BIS: Body Image Scale
BMI: Body mass index
BPI: Brief Pain Inventory
BPNSFS: Basic Psychological Need Satisfaction and Frustration Scale
BREQ-3: Behavioral Regulation for Exercise Questionnaire
CAML: Centro Académico de Medicina de Lisboa
CG: Control group
CHLO: Centro Hospitalar Lisboa Ocidental
CIDEFES: Centro de Investigação em Desporto, Educação Física, Exercício e Saúde
CTCAE v.5: Common Terminology Criteria for Adverse Events
CUF: Unidades Hospitalares CUF
ARE: Affective response to exercise
EORTC QLQ-C30: European Organization for Research and Treatment of Cancer Quality of Life Questionnaire Core 30
EORTC QLQ-BR23: European Organization for Research and Treatment of Cancer Quality of Life Questionnaire Breast Cancer Module
EORTC QLQ-BR45: European Organization for Research and Treatment of Cancer Quality of Life Questionnaire Breast Cancer Module
ESES: Bandura’s Exercise Self-Efficacy Scale
ExG: Structured exercise program
FEFD: Faculdade de Educação Física e Desporto
FS: Feeling Scale
HADS: Hospital Anxiety and Depression Scale
HFF: Hospital Fernando da Fonseca
IPAQ: International Physical Activity Questionnaire
IPOLFG: Instituto Português de Oncologia—Lisboa
LPA: Light physical activity
MD [95% CI]: Mean difference [95% confidence interval]
MVPA: Moderate-vigorous physical activity
NIH: National Institute of Health
PA: Physical activity
PAC: Brief physical activity counseling
PDI: Pain Disability Index
PSQI: Pittsburgh sleep quality index
RCT: Randomized controlled trial
RM: Repetition maximum
SDT: Self-Determination Theory
