# Supplementary material for: Effectiveness of supervised vs brief counselling physical activity promotion interventions in breast cancer survivors on aromatase inhibitors: the PAC-WOMAN randomized controlled trial
Source: Int J Behav Nutr Phys Act. 2026 Jan 22;23:13. doi: 10.1186/s12966-026-01873-5 (PMC12910743; doi:10.1186/s12966-026-01873-5)
Supplement: Supplementary file 2 — Supplementary Material 2 [file 12966_2026_1873_MOESM2_ESM.docx]

**Table S1: Exercise Program – mesocycles and session components; main BCTs**

| **Mesocycle** | | **Session Components** | **Type** | **Volume** | | **Intensity** |
| --- | --- | --- | --- | --- | --- | --- |
| **1** | 4 weeks | Warm-up | Mobility exercises | 6 exercises | 15-20 minutes | 3-4 RPEa |
|  |  |  |  | 1 min per exercise |  |  |
|  | 6 exercise sessions | Strength Training | Muscle Activation | 7 exercises | 30-45 minutes | 3-5 RPEa |
|  |  |  | Push | 2 sets |  |  |
|  |  |  | Pull | 15 Reps |  |  |
|  | 2 Theme Sessions: Movement and Pilates |  | Squat | 1 min rest period |  |  |
|  |  | Cardiovascular Training | Treadmill | 2 exercises | 20-25 minutes | 3-5 RPEa |
|  |  |  | Bicycle |  |  |  |
|  |  |  | Rowing machine |  |  | 40 - 45% HRR |
|  |  |  | Elliptical |  |  |  |
|  |  | Cool Down | Relaxation exercises | 5 minutes | | 1-2 RPEa |
| **2** | 4 weeks | Warm-up | Mobility exercises | 4 exercises | 5-7 minutes | 3-4 RPEa |
|  |  |  |  | 1 min per exercise |  |  |
|  | 6 exercise sessions |  | Muscle Activation | 3 exercises | 5-7 minutes |  |
|  |  |  |  | 2 series |  | 3-5 RPEa |
|  |  |  |  | 15 Reps |  |  |
|  |  |  |  | 30 min rest period |  |  |
|  | 2 Theme Sessions: Pilates and Aerobics | Strength Training | Push | 6 exercises | 30-40 minutes | 4-6 RPEa |
|  |  |  | Pull | 2 sets |  |  |
|  |  |  | Squat | 10-15 Reps |  | 60 – 70% 1-RM |
|  |  |  | Lunge | 1 min rest period |  |  |
|  |  | Cardiovascular Training | Treadmill | 2 exercises | 25-30 minutes | 4-6 RPEa |
|  |  |  | Bicycle |  |  |  |
|  |  |  | Rowing machine |  |  | 45 - 50% HRR |
|  |  |  | Elliptical |  |  |  |
|  |  | Cool Down | Relaxation exercises | 5 minutes | | 1-2 RPEa |
| **3** | 4 weeks | Warm-up | Mobility exercises | 2 exercises | 3-5 minutes | 3-4 RPEa |
|  |  |  |  | 1 min per exercise |  |  |
|  | 6 exercise sessions |  | Muscle Activation | 3 exercises | 5-7 minutes | 3-5 RPEa |
|  |  |  |  | 2 series |  |  |
|  |  |  |  | 15 Reps |  |  |
|  | 2 Theme Sessions: Flow and Dance |  |  | 30 min rest period |  |  |
|  |  | Strength Training | Push | 7 exercises | 30-45 minutes | 4-7 RPEa |
|  |  |  | Pull | 2-3 sets |  |  |
|  |  |  | Squat | 10-15 Reps |  | 60 – 70% 1-RM |
|  |  |  | Lunge | 1 min rest period |  |  |
|  |  | Cardiovascular Training | Treadmill | 2 exercises | 25-30 minutes | 4-7 RPEa |
|  |  |  | Bicycle |  |  |  |
|  |  |  | Rowing machine |  |  | 45 - 55% HRR |
|  |  |  | Elliptical |  |  |  |
|  |  | Cool Down | Relaxation exercises | 5 minutes | | 1-2 RPEa |
| **4** | 4 weeks | Warm-up | Mobility exercises | 2 exercises | 3-5 minutes | 3-4 RPEa |
|  |  |  |  | 1 min per exercise |  |  |
|  | 6 exercise sessions |  | Muscle Activation | 3 exercises | 5-7 minutes | 3-5 RPEa |
|  |  |  |  | 2 series |  |  |
|  |  |  |  | 15 Reps |  |  |
|  | 2 Theme Sessions: PAC Combat and Portuguese Traditional Games |  |  | 30 min rest period |  |  |
|  |  | Strength Training | Push | 7 exercises | 30-45 minutes | 5-8 RPEa |
|  |  |  | Pull | 2-3 sets |  |  |
|  |  |  | Squat | 10-15 Reps |  | 70 – 80% 1-RM |
|  |  |  | Lunge | 1 min rest period |  |  |
|  |  | Cardiovascular Training | Treadmill | 2 exercises | 25-30 minutes | 5-8 RPEa |
|  |  |  | Bicycle |  |  |  |
|  |  |  | Rowing machine |  |  | 55- 60% HRR |
|  |  |  | Elliptical |  |  |  |
|  |  | Cool Down | Relaxation exercises | 5 minutes | | 1-2 RPEa |
| **Behavioral Change Techniques (BCTs)*** | | 2.2. Feedback on behaviour \| 2.6. Biofeedback \| 3.2. Social support (practical) \| 4.1. Instruction on how to perform behaviour \| 6.1. Demonstration of the behaviour \| 15.1. Verbal persuasion about capability | | | | |

*Note: RPEa= Adapted Rated Perceived Exertion Scale; 1-RM= One-repetition maximum; Reps= repetitions; min= minutes | * BCTs used throughout the four mesocycles*

**Table S2: PAC Program – session content, motivational climate, and (M)BCTs**

| **Session Number** | **Session Themes** | **Intervention climate designed to support self-determination through the satisfaction of Basic Psychological Needs** | **Basic Psychological Need** | **Behavioral Change Techniques (BCTs)** | **Motivational Behavioral Change Techniques (MBCTs)** |  |
| --- | --- | --- | --- | --- | --- | --- |
| 1 | Reasons for change and related types of motivation; importance of self-monitoring strategies |  | Autonomy | 1.1 Goal setting (behavior) 1.2 Problem solving/coping planning 1.3 Goal setting (outcome) 1.5 Review behavior goal(s) 4.1 Instruction on how to perform a behavior 5.1 Health consequences 6.1 Modeling of the behavior 7.1 Prompts/cues 9.1 Persuasive argument 11.2 Regulate negative emotions 13.2 Reframing 13.4 Self-affirmation | 1. Elicit perspectives on condition or behavior 2. Prompt identification of sources of pressure for behavior change 5. Provide a meaningful rationale 6. Provide choice 7. Encourage the person to experiment and self-initiate the behavior 12. Use empathic listening 17. Assist in setting optimal challenge 21. Explore ways of dealing with pressure |  |
|  |  |  |  |  |  |  |
| 2 | Benefits of physical activity; types of physical activity and sedentary behaviors; SMART goal setting |  |  |  |  |  |
|  |  |  |  |  |  |  |
|  |  |  |  |  |  |  |
| 3 | Exercising safely and independently at home |  | Competence | 1.1 Goal setting (behavior) 1.2 Problem solving/coping planning 1.3 Goal setting (outcome)  1.4 Action planning 1.6 Discrepancy between current behavior and goal standard 2.3 Self-monitoring of behavior 3.1 Social support (general) 4.1 Instruction on how to perform a behavior 6.1 Modeling of the behavior 8.1 Behavioral rehearsal/practice 8.7 Graded tasks 12.1 Restructuring the physical environment 12.2 Restructuring the social environment 12.3 Avoidance/changing exposure to cues for the behavior | 6. Provide choice 7. Encourage the person to experiment and self-initiate the behavior 10. Show unconditional regard 14. Prompt identification and seek available social support 15. Address obstacles for change 17. Assist in setting optimal challenge 19. Help develop a clear and concrete plan of action 20. Promote self-monitoring 21. Explore ways of dealing with pressure |  |
| 4 | How to integrate physically active behaviors in daily life; importance of social support |  |  |  |  |  |
|  |  |  |  |  |  |  |
| 5 | Barriers and facilitating factors for including physical activity in daily routines; planning and dealing with barriers and setbacks; action and coping planning |  |  |  |  |  |
|  |  |  |  |  |  |  |
|  |  |  | Relatedness | 3.1 Social support (general) 6.1 Modeling of the behavior 6.2 Social comparison | 6. Provide choice 7. Encourage the person to experiment and self-initiate the behavior 8. Acknowledge and respect perspectives and feelings 10. Show unconditional regard 11. Demonstrate/show interest in the person 12. Use empathic listening 14. Prompt identification and seek available social support |  |
| 6 | Medical factors in managing cancer adverse events and physical activity |  |  |  |  |  |
| 7 | Boosting body image, self-acceptance, and their association with physical activity |  |  |  |  |  |
| 8 | Reassessing goals and long-term action plans |  |  |  |  |  |
